# AKT5 is a bona fide potassium channel and controls petiole growth in *Arabidopsis*

**DOI:** 10.1126/sciadv.aeh7630

**Published:** 2026-09-02

**Authors:** Yuki Muraoka, Shunsuke Kobayashi, Lisa Oskam, Michihiro Tateyama, Ayumu Masago, Yoshikazu Tanaka, Takeshi Yokoyama, Tadaomi Furuta, Hinano Takase, Ellen Tanudjaja, Shota Terashima, Haruto Shimizukawa, Taro Yamanashi, Shunya Saito, Hiroki Ijima, Miyuki Tanaka, Atsushi Miyamoto, Megumi Kato, Kanane Sato, Masaru Tsujii, Taishi Umezawa, Francisco J. Quintero, Francisco Rubio, Jörg Kudla, Masaaki Ito, Yoshihiro Kubo, Yasuhiro Ishimaru, Ronald Pierik, Nobuyuki Uozumi

**Affiliations:** ^1^ Department of Biomolecular Engineering, Graduate School of Engineering, Tohoku University; Sendai, 980-8579, Japan.; ^2^ Laboratory of Molecular Biology, Wageningen University and Research; Wageningen, 6708 TB, The Netherlands.; ^3^ Division of Biophysics and Neurobiology, Department of Molecular and Cellular Physiology, National Institute for Physiological Sciences; Okazaki, 444-8585, Japan.; ^4^ Department of Molecular and Chemical Life Sciences, Graduate School of Life Sciences, Tohoku University; Sendai, 980-8577, Japan.; ^5^ School of Life Science and Technology, Institute of Science Tokyo; Yokohama, 226-8501, Japan.; ^6^ Graduate School of Bio-Applications and Systems Engineering, Tokyo University of Agriculture and Technology; Koganei, 184-8588, Japan.; ^7^ Institute of Plant Biochemistry and Photosynthesis, Consejo Superior de Investigaciones Cientificas and University of Seville; Seville, 41092, Spain.; ^8^ Departamento de Nutrición Vegetal, CEBAS-CSIC, Campus de Espinardo; Murcia, 30100, Spain.; ^9^ Institut für Biologie und Biotechnologie der Pflanzen, Universität Münster; Münster, 48149, Germany.; ^10^ Department of Bioresources Engineering, National Institute of Technology, Okinawa College; Nago, 905-2192, Japan.

## Abstract

Potassium ion (K^+^) is essential for plant growth and development. Despite decades of study, the *Shaker*-type K^+^ channel AKT5 has remained functionally unassigned. Here, we report that AKT5 functions as a bona fide voltage-dependent K^+^ channel and serves as an essential regulator of petiole elongation. Structural analyses of AKT5 in a closed state and the AKT5-D403 variant in a pre-open state provide direct structural evidence for functional K^+^ channel activity. AKT5 assembled into a canonical tetramer with two-fold symmetry and, upon phosphorylation, operated as an inward-rectifying K^+^ channel activated at strongly hyperpolarized membrane potentials. AKT5 was predominantly expressed in young petioles, and loss of AKT5 function largely suppressed petiole elongation throughout the diurnal cycle, resulting in compact rosettes and reduced biomass under crowded growth conditions. These findings show that AKT5 is involved in a regulator of plant competitive growth and high-density performance.

## INTRODUCTION

Potassium is a major intracellular element in most organisms and one of three primary nutrients, along with nitrogen and phosphorus, required in large amounts by plants. Potassium in its ionic form (K^+^) is fundamental to plant growth responses that rely on turgor-driven cell expansion (*1*–*3*). Such responses underlie the elongation of various plant organs, including roots, stems, and petioles, allowing plants to adjust their architecture to surrounding environmental conditions. The growth response is influenced by external environmental conditions, such as changes in light availability and physical interactions with surrounding plants. These cues promote signaling processes that ultimately lead to cell elongation in specific tissues (*4*–*6*). K^+^ transporters and channels are fundamentally involved in regulating cellular osmotic potential and turgor during these adaptive growth processes. However, not all of them have been identified.

**Fig. 1.**
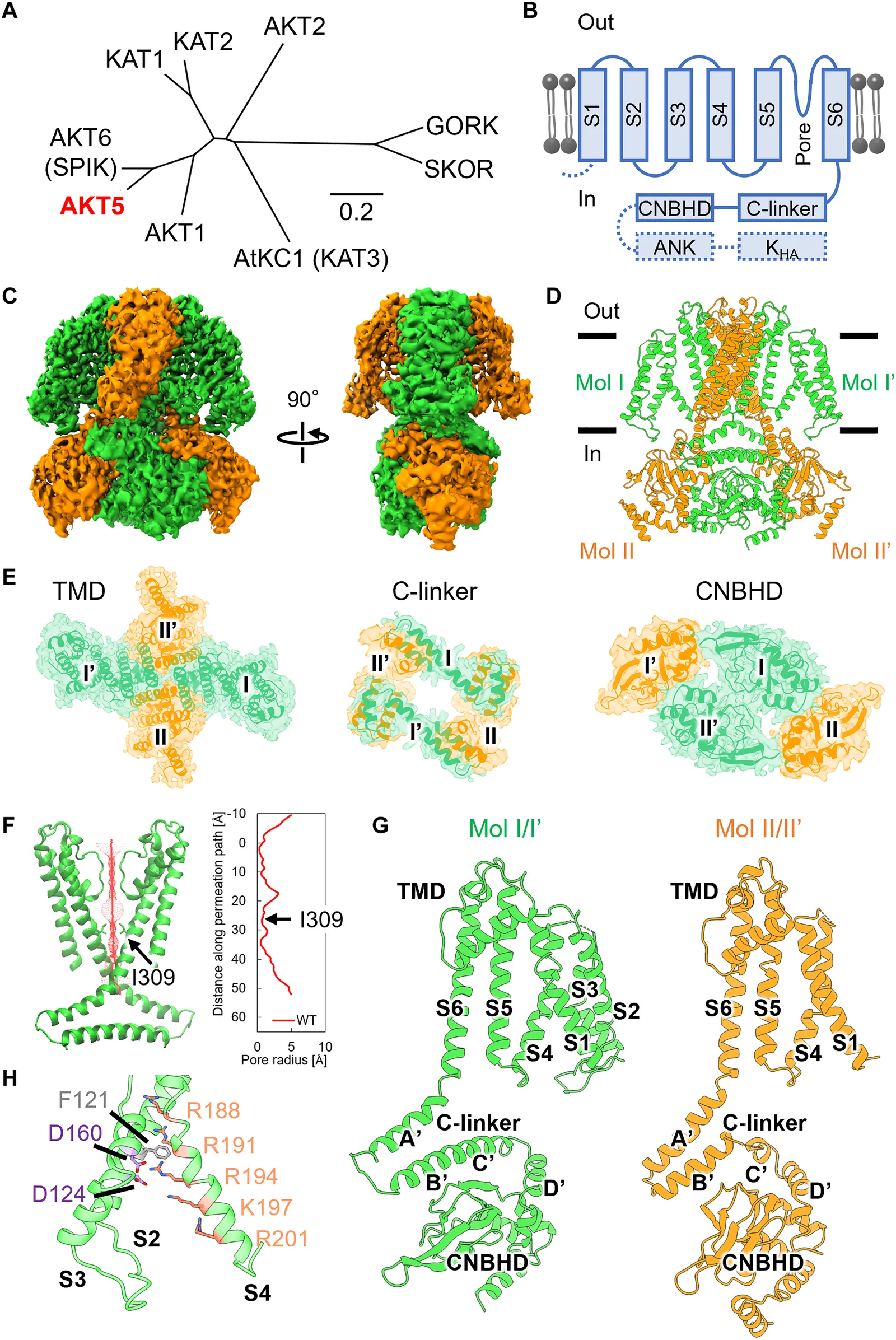
Cryo-EM reveals cytoplasmic C2 symmetry structure of the closed state of AKT5. (**A**) Phylogenetic tree of nine *Arabidopsis Shaker*-type K^+^ channels based on their amino acid sequences. The scale denotes substitutions per site. (**B**) Diagram depicting the major domains in each AKT5 subunit. Dashed lines indicate unresolved domains in our cryo-EM analysis. (**C**) Cryo-EM density map of AKT5 in lateral view. The four subunits are designated as Mol I/I′ (green) and Mol II/II’ (orange) according to their structural similarity to AKT1. (**D**) Reconstructed protein structure of AKT5. (**E**) Domain layers viewed sectionally from the intracellular side. (**F**) Radius of the pore region calculated by the HOLE program. Note that the Ile309 side chains (shown as sticks) restrict the inner gate. (**G**) Different conformations of Mol I/I′ (left) and Mol II/II’ (right). (**H**) Side view of the VSD and amino acids comprising the gating charge transfer center of Mol I/I′.

Since the identification of the *Shaker* K^+^ channel gene from Drosophila in 1987 (*7*), it has been recognized that *Shaker*-type, voltage-dependent K^+^ channels are widely present in both eukaryotic and prokaryotic cells, and many of their respective functions and physiological roles have been elucidated. In plants, there are different types of K^+^ channels/transporters with a range of functions (*8*, *9*). *AKT1* and *KAT1* were the first K^+^ channel genes isolated from plants and exhibit sequence homology to the *Shaker* K^+^ channel gene of Drosophila (*10*, *11*). The *Arabidopsis* genome encodes nine *Shaker*-type homologs (Fig. 1A), and most of their functions and physiological importance have been reported to date (*12*–*14*). AKT1, AtKC1 and SKOR regulate the absorption and distribution of K^+^ in the root (*15*–*17*), KAT1, KAT2 and GORK control stomatal movement (*18*–*20*), AKT2 regulates the transport of photosynthetic products in the phloem (*21*, *22*), and SPIK (also known as AKT6) is involved in pollen tube elongation (*23*). These eight K^+^ channels can be classified into four main groups based on their transport properties: KAT1, KAT2, AKT1, and AKT6 are inward-rectifying channels activated by membrane hyperpolarization (*23*–*26*); SKOR and GORK are outward-rectifying channels activated by membrane depolarization (*17*, *27*); AKT2 is a weakly rectifying channel (*28*–*30*); and AtKC1 is electrically silent, showing no K^+^ transport activity by itself (*31*). However, after more than 30 years of research, AKT5 (AT4G32500) remains the only member for which K^+^ transport activity or physiological function has not been reported.

The *Arabidopsis Shaker*-type K^+^ channels contain three regions: an intracellular N-terminal region, a transmembrane region, and an intracellular C-terminal region (*32*). The transmembrane region is composed of six helices (S1–S6), with S1–S4 forming the voltage sensing domain (VSD) and S5-S6 forming the pore domain (PD). The C-terminal region is further divided into five subdomains: (a) C-linker, (b) a cyclic nucleotide-binding homology domain (CNBHD), (c) a connection between CNBHD and the ankyrin repeat domain (ANK), termed the CNBHD-ankyrin bridge (*33*), (d) an ANK domain, and (e) a hydrophobic and acidic domain (K_HA_) (Fig. 1B). Interestingly, KAT1, KAT2 and AtKC1 do not have ANK. Recent advances in single-particle cryo–electron microscopy (cryo-EM) analysis have enabled the determination of the structures of KAT1, AKT1, SKOR, and GORK (*33*–*40*). A structural feature of the three ANK-containing channels (AKT1, SKOR and GORK) is that while the transmembrane regions exhibit almost four-fold (C4) symmetry, the cytoplasmic domains possess two-fold (C2) symmetry. For AKT1, it has been reported that a constitutively-active variant, AKT1^D403A^ (D403A), possesses C4 symmetry in its cytosolic domain, leading to the assertion that the transition from C2 to C4 symmetry is important for activation (*37*). On the other hand, the ANK-lacking KAT1 has C4 symmetry in its overall structure.

In this study, we demonstrate that AKT5 has a C2-symmetric cytoplasmic structure very similar to AKT1 and functions as an inward-rectifying K^+^ channel stimulated by kinase complexes composed of calcineurin B-like proteins (CBLs) and CBL-interacting protein kinases (CIPKs). We also show that AKT5 has a physiological function in regulating petiole growth in *Arabidopsis* plants. AKT5 is involved in vertical leaf movements and petiole elongation which are important growth responses that are regulated by light quality during canopy shading. This study fills in the “last piece”, which had been a mystery for more than 30 years, of the plant *Shaker*-type K^+^ channel family and provides important insights for a comprehensive understanding of K^+^ transporter function in plants.

## RESULTS

### Cryo-EM structure of AKT5

To explore the function of AKT5, we determined the structure at 3.62 Å resolution using cryo-EM (Fig. 1, figs. S1 and S2, and table S1). The resulting cryo-EM density map revealed a canonical tetrameric structure with two pairs of diagonal subunits, Mol I/I′ and Mol II/II’, similar to the conformation of AKT1 and GORK (Fig. 1, C and D) (*33*, *37*). The density map included a transmembrane domain (TMD), followed by the C-linker and CNBHD region. The cytosolic regions containing the ANK and K_HA_ domains were poorly resolved (Fig. 1, B to D). The TMD density map of Mol I/I′ was well-resolved throughout segments S1-S6, whereas in Mol II/II’, the map density in the S2-S3 region was poorly defined, preventing structural determination of this region (fig. S2). The TMD exhibited C4-like (pseudo-C4) symmetry, while the intracellular C-linker and CNBHD showed C2 symmetry (Fig. 1E). Based on calculations using HOLE (*41*), this structure represents the closed state, as the narrowest constriction was formed by Ile309 (Fig. 1F). We compared the AKT5 structure with the previously determined structure of *Arabidopsis Shaker*-type K^+^ channels (AKT1 (*37*), KAT1 (*34*), SKOR (*38*) and GORK (*39*)) using root mean square deviation (RMSD) between Mol I structures (fig. S3). The RMSD between AKT5 and AKT1 was the smallest (RMSD = 1.197 Å), indicating high structural similarity. Comparison with SKOR and GORK also resulted in relatively small RMSD values, while comparison with KAT1 had the largest value (3.328 Å, fig. S3). This result is consistent with the symmetry differences, where AKT5, AKT1, SKOR and GORK adopt a C2 symmetry in the cytoplasmic region, while KAT1 forms a C4 symmetry. The C-linker of AKT5 comprises four α-helices (A’-D′). In Mol I/I′, the B′ and C′ helices were arranged linearly, while in Mol II/II’, they adopted a ‘kinked’ conformation, which is also observed in AKT1 (*36*, *37*) (Fig. 1G). Within the VSD, a regularly spaced sequence of five positively charged residues in the S4 helix (Arg188, Arg191, Arg194, Lys197 and Arg201) seems to contribute to the gating charge movements. Phe121 and Asp124 in S2, and Asp160 in S3 likely form a constriction, the so-called “gating charge transfer center”, around which the S4 residues pivot (Fig. 1H) (*42*). We observed residues Arg188 and Arg191 to locate above, and Arg194, Lys197 and Arg201 below the charge transfer center. This arrangement is consistent with the ‘up’ position of the VSD as expected of the channel when isolated in the absence of an electrical field (*37*, *39*). The overall structural data suggest that AKT5 forms an inward-rectifying K^+^ channel.

### Intermolecular interaction around Asp403 restricts AKT5 activation

**Fig. 2.**
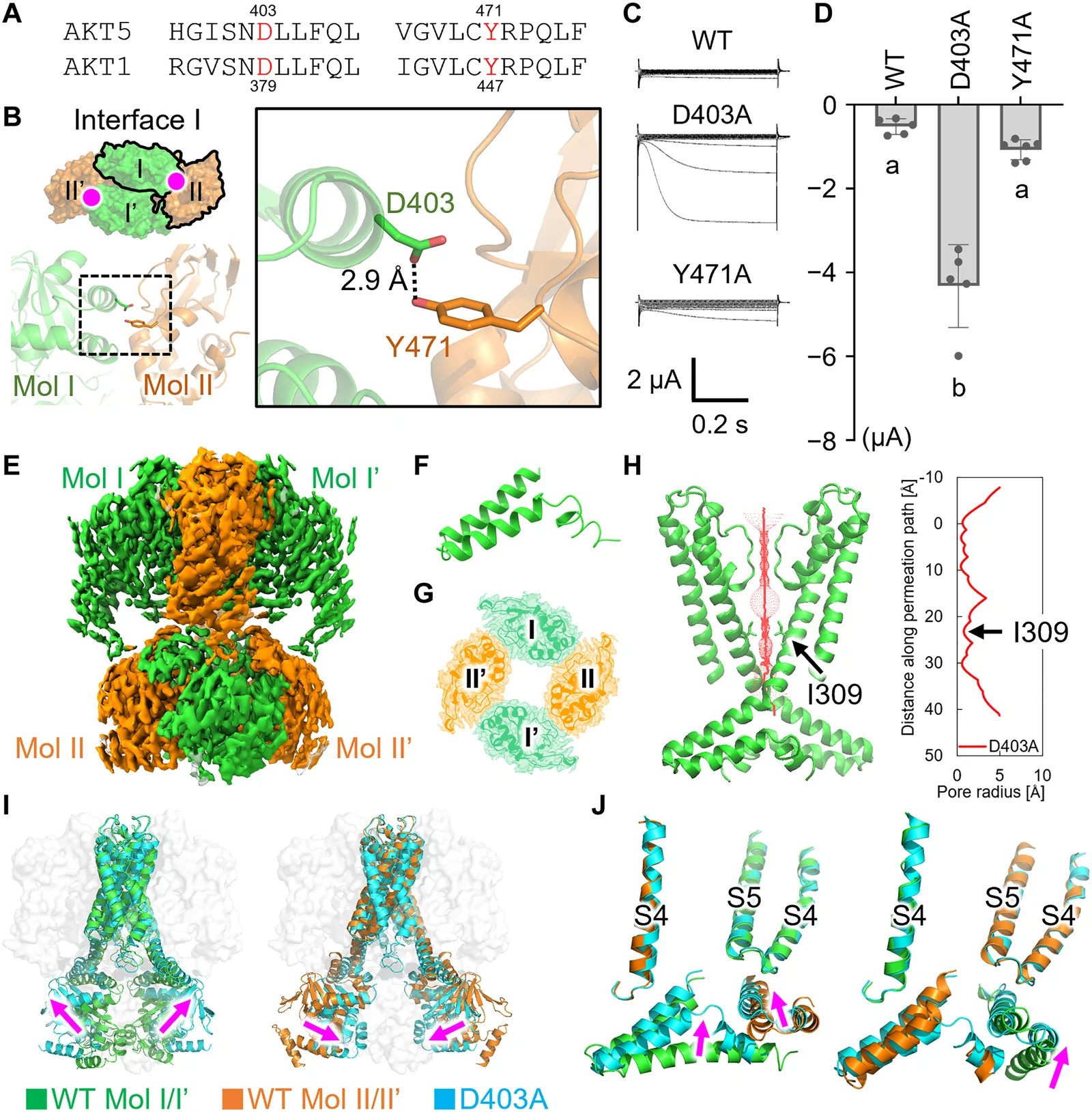
Cryo-EM structure of the pre-open state AKT5 variant. (**A**) Amino acid sequence alignment between AKT5 and AKT1 in regions critical for dimer interfaces. The interacting residues at the interfaces are shown in red. (**B**) The packing interface in CNBHD of AKT5 (top left). The boxed region in the left lower panel is enlarged on the right. Asp403 from Mol I/I′ and Tyr471 from Mol II/II’ form a hydrogen bond. (**C**) Representative current profile of *Xenopus* oocytes expressing wild-type AKT5 (WT) or the AKT5^D403A^ and AKT5^Y471A^ variants, in which the interactions were disrupted by substitution with Ala. Currents were elicited by voltage steps from +10 to −190 mV (Δ = −20 mV). Currents were recorded in the buffer containing 120 mM K^+^. Images were cropped to show only the relevant regions. (**D**) Steady-state currents of WT, AKT5^D403A^ and AKT5^Y471A^ recorded at −190 mV as shown in panel (C). The data are presented as mean ± SD (*n* = 5–6). Different letters indicate significant differences (ordinary one-way ANOVA with Tukey’s multiple comparisons test; *P* < 0.05). (**E**) Structure of AKT5^D403A^. The tetramer assembles with C4 symmetry. Details of the cryo-EM data processing are provided in fig. S4B. (**F**) The C-linker in AKT5^D403A^ undergoes a dramatic conformational change into the canonical ‘kinked’ conformation. (**G**) Sectional view of the C4 symmetry of the CNBHD layer of AKT5^D403A^ from the intracellular side. (**H**) Radius of the pore region calculated by the HOLE program. Ile309 restricts the inner gate (stick). (**I** and **J**) Structural comparison of AKT5 and AKT5^D403A^. CNBHDs undergo a dramatic conformational change associated with the symmetry rearrangement (I). Conformational changes of C-linkers associated with the symmetry rearrangement (J). Arrows indicate the direction of displacement of AKT5^D403A^ relative to the wild-type AKT5.

The structural similarity to AKT1 suggested that AKT5 activation likely involves a transition from C2 symmetry (closed state) to C4 symmetry (open state) in the cytoplasmic region. In AKT1, this transition occurs upon disruption of hydrogen bonds between Asp379 and Tyr447, both located within CNBHD (*37*), and the corresponding residues in AKT5 are Asp403 and Tyr471 (Fig. 2, A and B). When these residues were replaced individually with Ala, and the activity of the resulting variants was determined by two-electrode voltage clamping in *Xenopus* oocytes, AKT5^D403A^ had an increased K^+^ current amplitude, while the AKT5^Y471A^ showed slightly higher activity compared to the wild type (WT, Fig. 2, C and D). The data suggest that AKT5 could function as a K^+^ channel, and the inter-subunit interactions around Asp403 restrict AKT5 activation.

AKT5^D403A^ was purified to homogeneity for cryo-EM analysis, and the structure was determined at 2.60 Å resolution (fig. S4 and table S1). The C4 symmetric cytoplasmic domain was clearly identified from the results of 2D classification (fig. S4B). In this structure, all four C-linkers adopted a canonical ‘kinked’ conformation (Fig. 2, E and F and fig. S5, A and B) and displayed C4 symmetry across the TMD, C-linker, and CNBHD regions (Fig. 2G and fig. S5C). The RMSD of AKT5^D403A^ relative to AKT1^D379A^ was 1.001, indicating highly similar structures (fig. S5D) (*37*). HOLE analysis revealed a closed gate (Fig. 2H) and the VSD adopted an ‘up’ conformation (fig. S5E). Compared to AKT5, from the side view, the CNBHDs of Mol I/I′ tilted up toward the membrane, whereas the CNBHDs of Mol II/II’ were close to the K^+^ permeation axis These conformational changes were coupled with the reorientation of C-linkers. The ‘straight’ helical structure of B’C’ in the C-linkers of Mol I/I’ in AKT5 broke into two short helices in AKT5^D403A^ (B’ and C’ helices). These findings imply that AKT5 adopts two distinct cytosolic conformations: a closed cytosolic C2-symmetric structure revealed by structural analysis of the wild-type AKT5 (Fig. 1), and a pre-open cytosolic C4-symmetric structure identified in AKT5^D403A^ (Fig. 2, E to J).

**Fig. 3.**
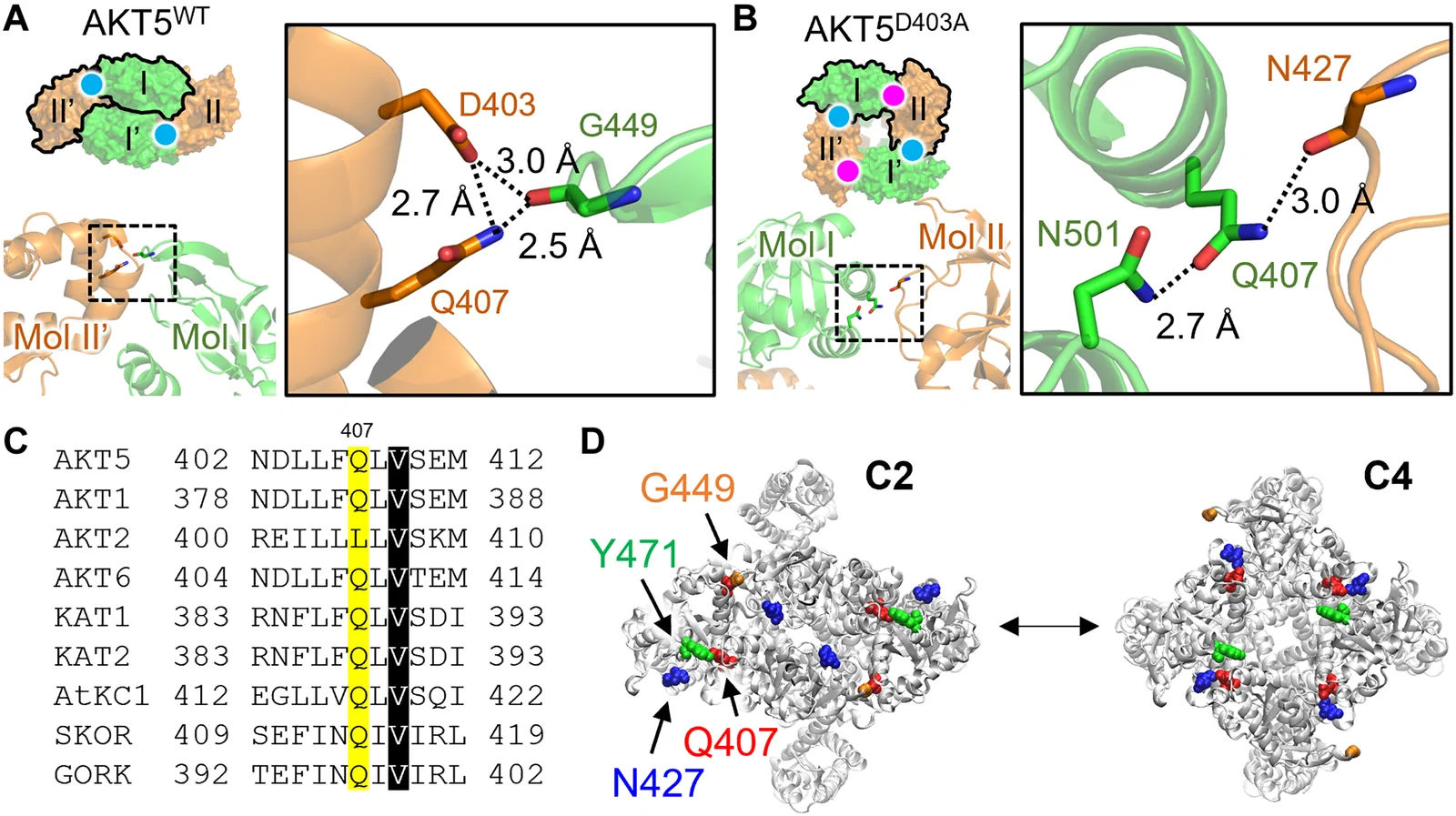
Gln407 mediates inter-subunit interactions at the CNBHD interface. (**A**) Gly449 from Mol I/I′, and Gln407 and Asp403 from Mol II’/II form tripartite interactions at the CNBHD interfaces of AKT5. (**B**) Asn427 from one protomer, and Gln407 and Asn501 from the adjacent protomer form tripartite interactions at the CNBHD interfaces of AKT5^D403A^. (**C**) Gln407 is highly conserved in all *Arabidopsis Shaker*-type channels, except for AKT2. (**D**) Comparison of interaction changes at the CNBHD interfaces between the C2- and C4-symmetric structures of AKT5. The model structures were generated based on the cryo-EM structures of AKT5 (PDB ID: 9X0B), representative for the C2-symmetric structure and AKT5^D403A^ (PDB ID: 9X0C), representative for the C4 symmetric structure.

In addition to the D403-Y471 interaction, we identified a previously unrecognized tripartite interaction network in both AKT5 and AKT5^D403A^ structures. In the wild-type AKT5, Asp403 and Gln407 in Mol II/II’ interacted with the main-chain carbonyl group of Gly449 in Mol I′/I (Fig. 3A). In AKT5^D403A^, Gln407 in Mol I/I′ created another hydrogen-bonding network with Asn501 within the same protomer and Asn427 from the adjacent subunit Mol II/II’ (Fig. 3B). Sequence alignment further revealed that Gln407 is conserved among nine *Arabidopsis Shaker*-type channels, with the exception of AKT2, implying a broadly conserved role in channel function (Fig. 3C). Together, these findings indicate that Gln407 participates in the stabilization of both C2- and C4-symmetric cytosolic conformations of AKT5, thereby playing a key role in conformational transitions underlying channel gating (Fig. 3D and movie S1).

### Rescue of K^+^ uptake-deficiency of a yeast mutant by AKT5 with CBL-CIPKs

**Fig. 4.**
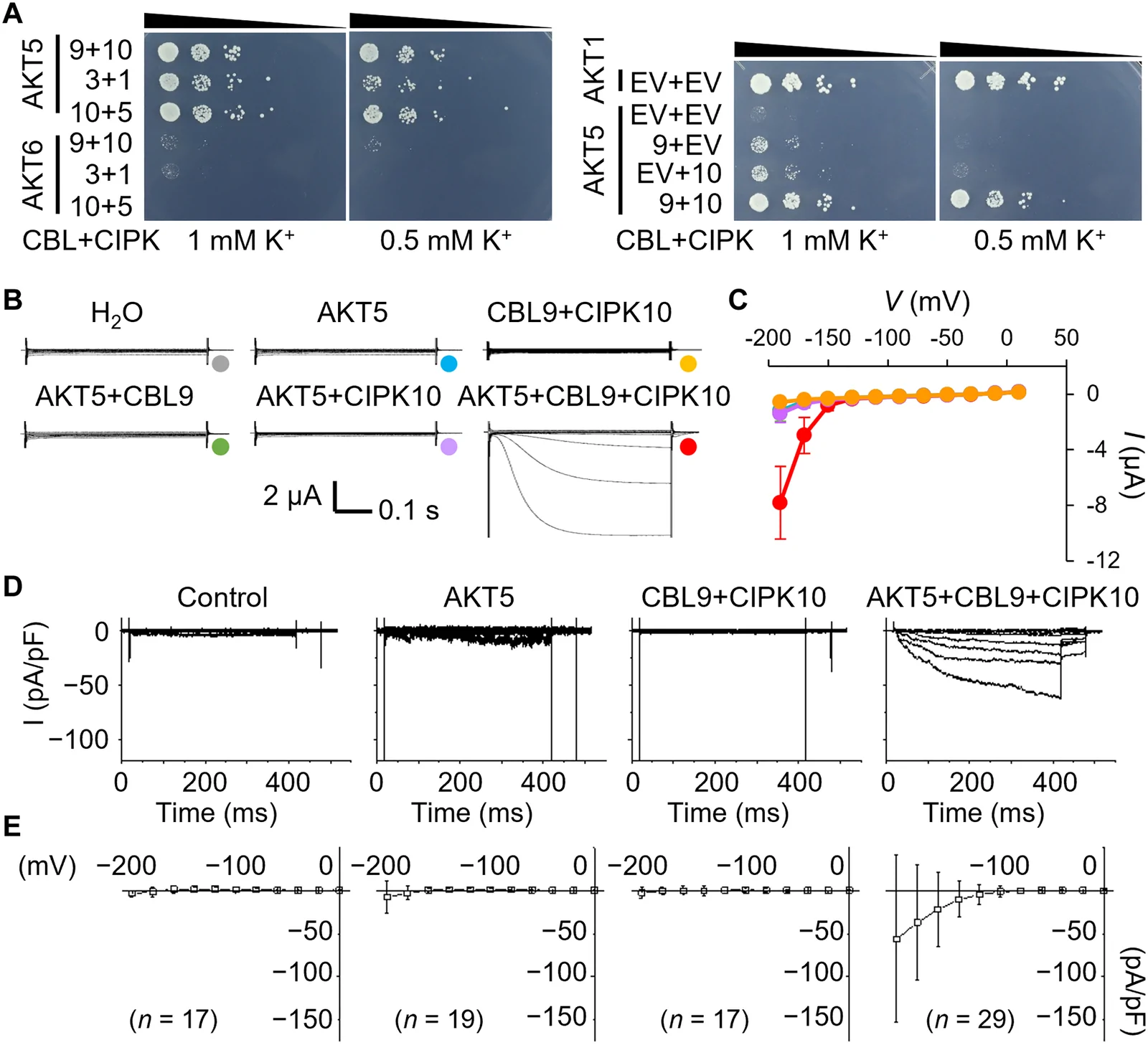
AKT5 functions as an inward-rectifying K^+^ channel. (**A**) Dilution series of *Saccharomyces cerevisiae* 9.3 strain transformed with AKT1, AKT5 and AKT6 and three pairs of CBLs-CIPKs, CBL9-EV (empty vector), EV-CIPK10 or EV-EV grown on the agar medium containing 1 mM or 0.5 mM K^+^. (**B**) Representative current profile of *Xenopus* oocytes expressing CBL9, CIPK10 or AKT5. Currents were elicited by voltage steps from +10 to −190 mV (Δ = −20 mV). Currents were recorded in the buffer containing 120 mM K^+^. Images were cropped to show only the relevant regions. (**C**) *I*-*V* relationship of steady-state currents in *Xenopus* oocytes versus applied voltages in (B). The data are presented as mean ± SD (*n* = 6). (**D** and **E**) Whole-cell patch-clamp recordings from COS-7 cells expressing CBL9, CIPK10 and/or AKT5. Currents were elicited by voltage steps from −200 to 0 mV (Δ = +20 mV). Images were cropped to display only the relevant regions. Representative macroscopic currents are shown in (D), and steady-state current densities are plotted as a function of the clamped membrane potential in (E) (mean ± SD). All cells were co-transfected with *YFP*.

The structural similarity of AKT5 and AKT1 suggests that activation of AKT5 may also be similar to activation of AKT1 by the pairs of CBL (Calcineurin B-like proteins) 1/9 and CIPK (CBL-interacting protein kinases) 23 (*43*). We conducted a yeast functional complementation screen to identify CBL-CIPK complexes responsible for AKT5 stimulation by testing every possible combination of the 26 CIPKs and 10 CBLs from *Arabidopsis*. These 260 pairs of CBL-CIPKs were co-expressed with AKT5 in the K^+^-uptake-deficient *Saccharomyces cerevisiae* 9.3 mutant (*44*). In total, yeasts transformed with 247 of the 260 combinations grew in K^+^-sufficient medium (0.5 mM K^+^), but 13 of the 260 CBL-CIPK-AKT5 expressing strains did not grow (fig. S6). Seventeen of the 247 transformants also showed robust growth in K^+^-limited medium (fig. S6). To corroborate the ability of these 17 pairs of CBL-CIPK to activate AKT5, an additional growth complementation assay was performed using a dilution series of yeast cultures on different K^+^ concentrations (fig. S7A to S7C). Nine of these CBL-CIPK pairs (CBL1–CIPK2, CBL2–CIPK11, CBL3–CIPK20, CBL6–CIPK16, CBL6–CIPK20, CBL7–CIPK23, CBL9–CIPK5, CBL9–CIPK9, and CBL10–CIPK9) complemented the growth phenotype of the yeast strain even without the presence of AKT5 and were therefore eliminated (fig. S7D). Out of the remaining eight combinations, we selected three that showed higher growth: CBL9-CIPK10-AKT5, CBL3-CIPK1-AKT5 and CBL10-CIPK5-AKT5 (Fig. 4A and fig. S7, A to C and E). All three pairs of CBL-CIPK with AKT6 failed to complement the K^+^ uptake deficiency, despite AKT6 being the closest homolog to AKT5 (76.8% identical residues; Figs. 1A, 4A, and fig. S7E). The data indicate that AKT5 can be activated by CBL9-CIPK10, CBL3-CIPK1 and CBL10-CIPK5.

### AKT5 activation depends on phosphorylation

We examined activation of AKT5 by the three CBL-CIPK pairs through two-electrode voltage clamp measurements in *Xenopus laevis* oocytes. Oocytes expressing AKT5 with CBL9-CIPK10, CBL3-CIPK1 and CBL10-CIPK5 exhibited increased current amplitudes when the membrane potential was hyperpolarized (Fig. 4, B and C and fig. S8A). It is known that AKT1 is activated by CBL1 + CIPK23 (*43*, *45*). Weak complementation by CBL1 + CIPK23 was also observed in yeast (fig. S6), prompting us to test this pair in *Xenopus* oocytes. CBL1 + CIPK23 activated AKT5 to a lesser extent compared to CBL9 + CIPK10 (fig. S8B and S8C). Hereafter, we focused on the CBL9-CIPK10 combination for further analysis. No apparent K^+^ current was observed when AKT5 was expressed alone, or when AKT5-CBL9, AKT5-CIPK10 or CBL9-CIPK10 were co-expressed (Fig. 4, B and C). The data indicate that CBL9-CIPK10 activated AKT5-mediated K^+^ channel activity. Application of 10 mM tetraethylammonium chloride (TEA), a classical K^+^ channel blocker, significantly reduced the currents in AKT5 + CBL9 + CIPK10-expressing oocytes (fig. S8, D and E), confirming that the observed currents are primarily mediated by AKT5. However, we cannot exclude the possibility that part of the recorded current may arise from endogenous oocyte currents. We also examined whether CBL9 and CIPK10 were required for AKT5 function in COS-7 cells by whole-cell patch-clamp recordings. No apparent K^+^ currents were observed in COS-7 cells expressing AKT5 alone (Fig. 4, D and E and fig. S8F). However, significant K^+^ currents were observed in COS-7 cells co-expressing AKT5 and CBL9-CIPK10 (Fig. 4, D and E and fig. S8F). In addition, no apparent K^+^ currents were detected in cells expressing CBL9-CIPK10 without AKT5 (Fig. 4, D and E and fig. S8F). These results provide strong evidence that AKT5 is a functional K^+^ channel.

**Fig. 5.**
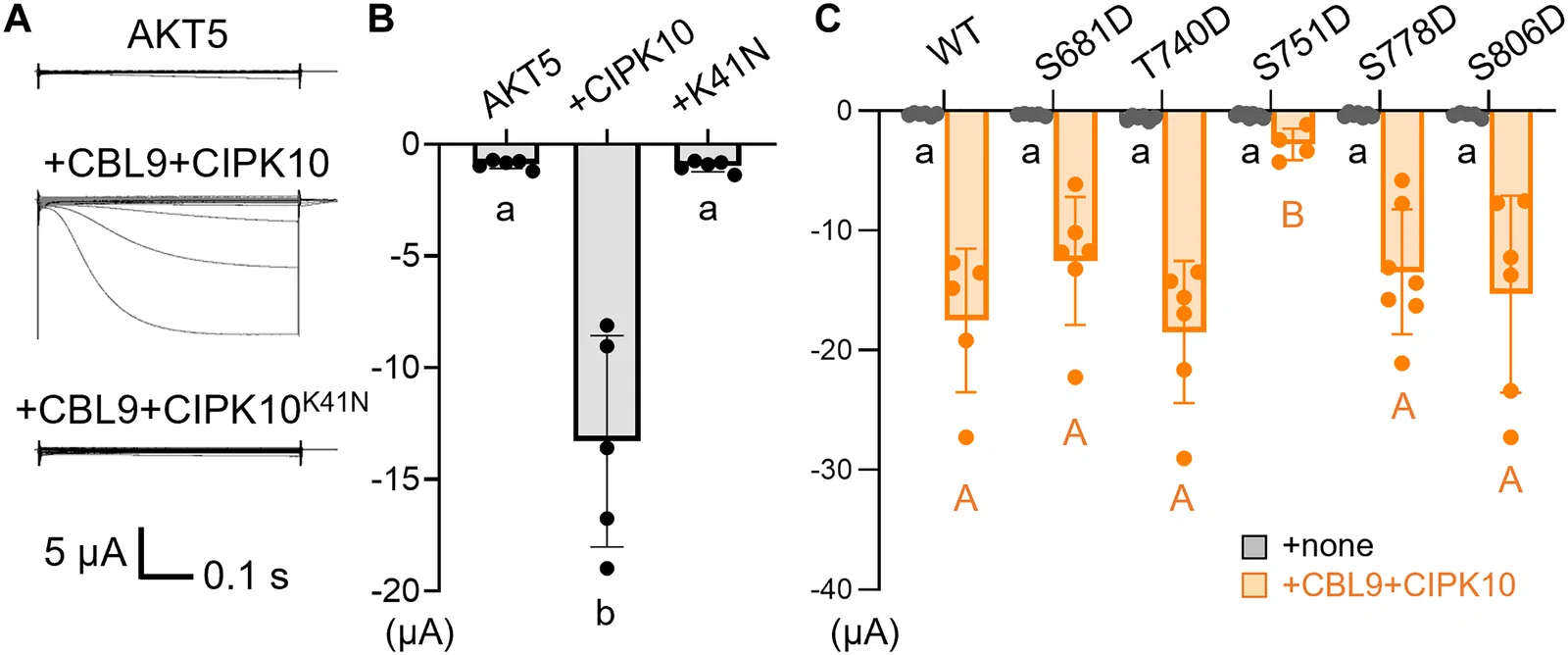
Activation of AKT5 by CBL9–CIPK10. (**A**) Representative current profile of *Xenopus* oocytes expressing AKT5 alone, AKT5 together with CBL9-CIPK10, or CBL9-CIPK10^K41N^. Currents were elicited by voltage steps from +10 to −190 mV (Δ = −20 mV). Images were cropped to show only the relevant regions. (**B**) Steady-state currents of AKT5 alone, AKT5 together with CBL9-CIPK10, or CBL9-CIPK10^K41N^ recorded at −190 mV as shown in panel (A). The data are presented as mean ± SD (*n* = 5). Different letters indicate significant differences (ordinary one-way ANOVA with Tukey’s multiple comparisons test; *P* < 0.05). (**C**) Steady-state currents recorded at −190 mV for AKT5 Asp-substituted variants (Ser/Thr to Asp) at phosphorylation sites identified by mass spectrometry, expressed in *Xenopus* oocytes. Data are presented as mean ± SD (*n* = 5–9). Different letters indicate statistically significant differences (one-way ANOVA with Tukey’s multiple-comparisons test; *P* < 0.05).

To examine whether AKT5 activation depends on phosphorylation by the CBL–CIPK complex or on other phosphorylation-independent mechanisms, we prepared a kinase-dead CIPK mutant. CIPK23 contains a conserved lysine, Lys60, within its ATP-binding site, and substitution of this residue with Asn (CIPK23^K60N^) abolishes the kinase activity, resulting in a marked loss of AKT1 activation capacity (*45*, *46*). The corresponding Lys at position 41 in CIPK10 was therefore converted to Asn to generate a kinase-inactive variant (CIPK10^K41N^). When CIPK10^K41N^ was co-injected with CBL9 and AKT5 into oocytes, no inward currents were detected at 24 hours, 48 hours, and 72 hours after injection (Fig. 5, A and B). These results suggest that phosphorylation by CIPKs is needed for AKT5 activation.

Phosphorylation of Ser26 and Ser338 by CBL1/CIPK23 is required for AKT1 activation (*37*). In AKT5, the corresponding residues, Ser43 and Ser362, are conserved (fig. S9A). To assess the functional relevance of these residues, we substituted them with Ala (mimicking the un-phosphorylated form) or Asp (pseudo-phosphorylated form). The activity of the single and double Ala variants (AKT5^S43A^, AKT5^S362A^ and AKT5^S43A/S362A^) and AKT5^S43D^ in the presence of CBL9-CIPK10 remained similar to that of wild-type AKT5 (fig. S9, B and C). The pseudo-phosphorylated Asp substitution variants, AKT5^S362D^ and AKT5^S43D/S362D^, had no detectable channel activity (fig. S9, B and C). The data suggest that the phosphorylation sites crucial for activation differ between AKT5 and AKT1.

To identify the phosphorylation sites of AKT5 targeted by CBL9-CIPK10, we incubated AKT5 protein purified from Sf9 with CBL9 and CIPK10 produced in a silkworm cell-free expression system (*47*), followed by mass spectrometry analysis. The analysis identified five phosphorylated residues, Ser681, Thr740, Ser751, Ser778, and Ser806 (fig. S10). Then, we replaced each of these five residues with either Ala or Asp and expressed them in *Xenopus* oocytes. The S681A/D, T740A/D, S778A/D and S806A/D variants showed CBL9-CIPK10-dependent activation similar to that of the wild-type AKT5 (Fig. 5C and fig. S11). These results implied that the phosphomimic AKT5 variants still required CBL9-CIPK10 for channel activity, and/or that Asp substitution does not fully reproduce the functional properties of phosphorylated Ser/Thr residues (Fig. 5C). Notably, CBL9-CIPK10-dependent activation was almost completely abolished in both AKT5^S751A^ and AKT5^S751D^ (Fig. 5C and fig. S11), suggesting that Ser751 is involved in CBL9-CIPK10-mediated regulation of AKT5 activity.

Given our finding that Gln407 contributes to the formation of the pre-open state (Fig. 3B), we assessed K^+^ channel activity of AKT5^Q407A^ in the presence of CBL9 and CIPK10 (fig. S12). In contrast to AKT5, AKT5^Q407A^ exhibited only low K^+^ channel activity, suggesting that Gln407 is crucial for the transition of AKT5 to the open state and that assistance of CBL9-CIPK10 is required.

### AKT5 affects petiole growth in *Arabidopsis thaliana*

**Fig. 6.**
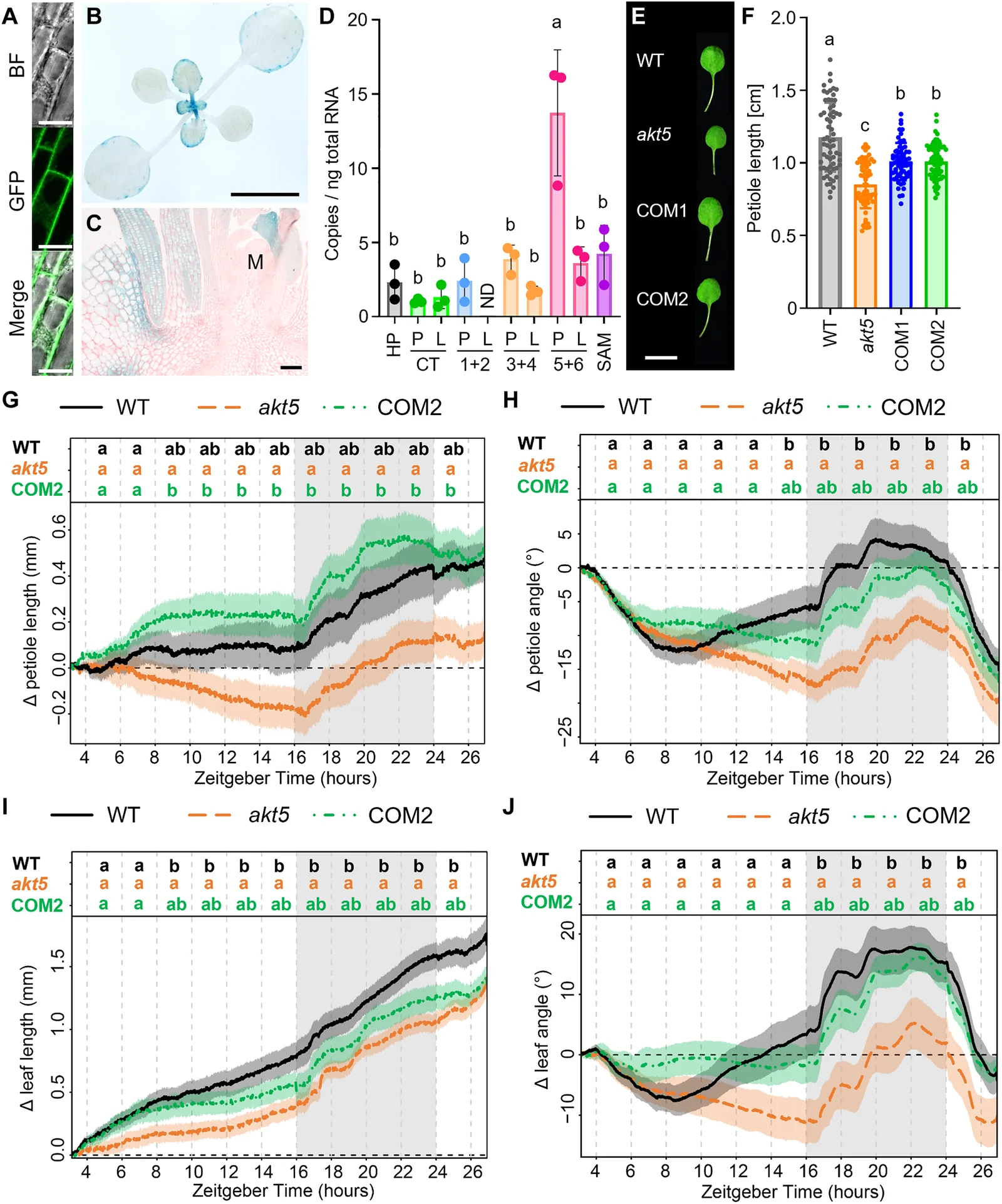
AKT5 regulates petiole growth and angle in *Arabidopsis thaliana*. (**A**) Plasma membrane localization of AKT5 in *Arabidopsis* plants. AKT5 fused to GFP in root cells (*P_35S_*-*GFP*-*AKT5*) was visualized by confocal microscopy. Scale bar, 20 μm. (**B** and **C**) Histochemical localization of *AKT5* expression in 3-week-old transgenic *Arabidopsis* plants expressing the GUS reporter genes under the control of the *AKT5* promoter (*P_AKT5_*-*GUS*). (B) Image of whole plant. Scale bar, 5 mm. (C) Cross-section of area around the shoot apical meristem (M). Scale bar, 100 μm. (**D**) Relative transcript levels of *AKT5* in the hypocotyl (HP), and petioles (P) and lamina (L) of the cotyledons (CT) and different leaf stages (leaves number 1–6), as well as in the shoot apical meristem (SAM), of 17-day-old *Arabidopsis* plants. Data are presented as mean ± SD (*n* = 3). Different letters indicate significant differences (ordinary one-way ANOVA with Tukey’s multiple comparisons test; *P* < 0.05). (**E**) Representative images of one of the first true paired leaves detached from 17-day-old plants grown in soil. WT, the wild-type plants; *akt5*, the *akt5* mutant; COM1 and COM2, two complementation lines expressing *P_AKT5_-GFP-AKT5* in the *akt5* background. Scale bar, 1 cm. (**F**) Quantification of petiole length of the 1st-4th true leaves. Data are presented as mean ± SD (*n* = 63–64). Different letters indicate significant differences (one-way ANOVA with Tukey’s multiple comparisons test; *P* < 0.05). (**G** to **J**) Time-course changes in petiole length (**G**), petiole angle (**H**), leaf length (**I**), and leaf angle (**J**) of leaf 3. Data are presented as mean ± SE (*n* = 16 for WT, 15 for the *akt5* mutant and 14 for COM2). Different letters indicate statistically significant differences (one-way ANOVA test with Tukey post hoc comparisons; *P* < 0.05).

**Fig. 7.**
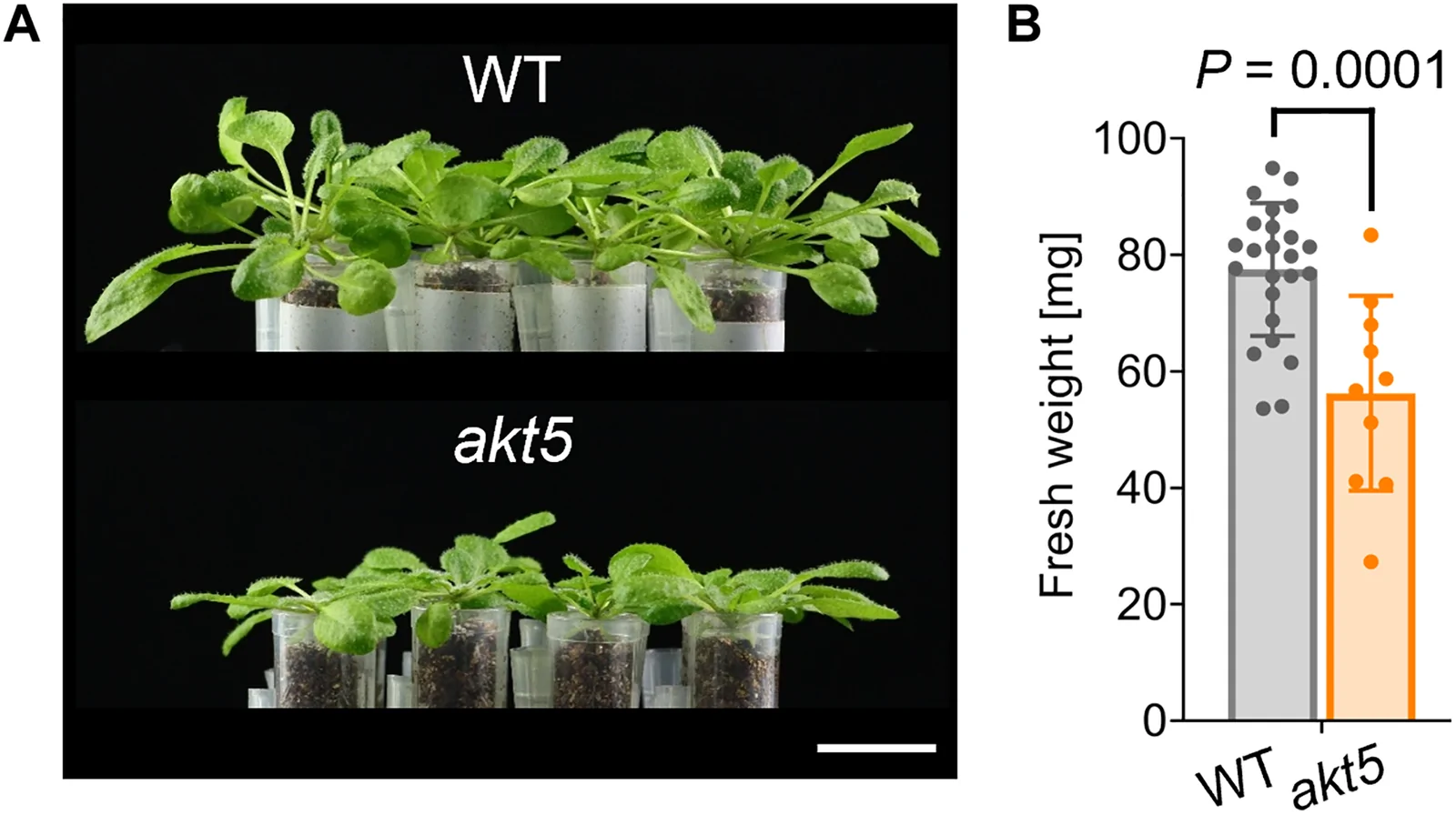
AKT5 contributes to plant growth in dense vegetation. (**A**) Representative images depicting the crowded growth conditions of WT (top), and the *akt5* mutant (bottom) growing in 4 ml tubes. Scale bar, 20 cm. (**B**) Fresh weight of WT and the *akt5* mutant shown in (A). Data are presented as mean ± SD (*n* = 24 for WT and *n* = 10 for the *akt5* mutant). Statistical significance was assessed using an unpaired two-tailed *t* test.

To explore the biological role of AKT5 in plants, we first examined its subcellular and tissue-specific localization. We generated *Arabidopsis* plants overexpressing AKT5 fused to GFP under control of the *35S* promoter (*P_35S_-GFP*-*AKT5*) and examined GFP fluorescence at the root tips, where chloroplast autofluorescence is minimal. The GFP signal was localized to the plasma membrane (Fig. 6A and fig. S13, A to C). To determine the tissue-specific expression pattern of *AKT5*, we generated transgenic *Arabidopsis* plants harboring the GUS reporter gene under the control of the *AKT5* promoter (*P_AKT5_-GUS*). Strong *GUS* staining was detected in young petioles (Fig. 6, B and C). Consistent with this result, RT-qPCR analysis revealed that *AKT5* transcript levels were significantly higher in the youngest petioles compared to mature petioles and other vegetative tissues (Fig. 6D). To examine the physiological function of AKT5 in petioles, we analyzed the phenotypes of the *akt5* mutant (SALK_015179C) and two complementation lines expressing *P_AKT5_*-*GFP*-*AKT5* in the *akt5* background (COM1 and COM2) (fig. S13D). The *akt5* mutant showed no substantial differences in root length (fig. S13, E and F) but exhibited reduced petiole elongation compared to AKT5 (Fig. 6, E and F). We confirmed that this phenotype was partially rescued in COM1 and COM2 plants (Fig. 6, E and F). Time-course analysis of petiole elongation and angle revealed a marked increase during the night period (ZT16–24) in the wild type (WT) and COM2, as previously reported for the wild type (*48*), whereas the length and angle of the petiole remained significantly lower in the *akt5* mutant (Fig. 6, G and H and movie S2). Similar phenotypic effects in the *akt5* mutant were observed in entire leaf, including both petiole and lamina, with significant differences in length and angle, compared to the wild type, and recovery in COM2 (Fig. 6, I and J and movie S2). Petiole elongation enhances light capture in plants under crowded conditions, increasing their competitive ability (*5*, *6*). To study the role of AKT5 in this aspect, we cultured the wild type or the *akt5* plants under crowded conditions. The fresh weight of the *akt5* mutant was significantly smaller compared to the wild type, suggesting that AKT5 indeed contributes to growth in dense vegetation (Fig. 7, A and B and fig. S13, G and H).

## DISCUSSION

AKT5 is the “last piece” of the nine *Shaker*-type K^+^ channels in *Arabidopsis* to be identified in terms of its functional and physiological characteristics. Here, we demonstrate that AKT5 functions as a key K^+^ channel that impacts petiole elongation. When expressed alone, AKT5 did not exhibit detectable K^+^ uptake activity in yeast, *Xenopus* oocytes and COS-7 cells (Fig. 3), which likely accounts for the lack of previous functional studies on AKT5. However, when Asp403 was replaced with Ala, AKT5^D403A^ adopted a C4-symmetric tetramer conformation, and a hyperpolarization-activated K^+^ current was observed (Fig. 2). Consequently, coexpression with one of at least three pairs of CBL-CIPK kinases resulted in activation of the K^+^ channel function of AKT5 (Fig. 3). These results suggest that an intracellular regulatory system like phosphorylation might stimulate AKT5 in planta.

Although AKT5 belongs to the *Shaker*-type K^+^ channel family and shares sequence similarity with AKT1, our structural analyses revealed that AKT5 exhibits both conserved and unique features that distinguish its gating behavior and biological function. A major difference between AKT1 and AKT5 lies in the way they are regulated by phosphorylation. While AKT1 activation depends on phosphorylation at Ser26 and Ser338, neither of the corresponding residues (Ser43 and Ser362) plays an essential role in AKT5. In contrast, we identified Ser751 as a critical phosphorylation site required for AKT5 activation by CBL–CIPK complexes (Fig. 5C). These results highlight a distinct phosphorylation code that differentiates AKT5 from AKT1 and likely reflects specialization of tissue-specific signaling inputs. Among all 260 CBL–CIPK combinations tested by yeast complementation screening, only a limited number of pairs were capable of activating AKT5, CBL9–CIPK10, CBL3–CIPK1, and CBL10–CIPK5 (Fig. 4A and fig. S7). Notably, the CBL9–CIPK10 combination failed to activate SPIK/AKT6 in both yeast and *Xenopus* oocytes, despite its high sequence similarity to AKT5 (Fig. 4A and fig. S8A). These findings support the existence of a channel-specific CBL–CIPK activation system, wherein individual *Shaker*-type channels are gated by distinct kinase modules, allowing diversification of electrical and signaling responses across different cellular contexts. These data are however based on heterologous expression systems, and the physiological relevance of specific CBL–CIPK complexes in planta remains to be determined.

Structural analyses of AKT5 and AKT5^D403A^ provided insights into both the closed and pre-open conformational states of AKT5 (Figs. 1 and 2). The interaction between Asp403 and Tyr471, an equivalent residue pair that is also conserved in AKT1 (*37*) (Fig. 2B), was crucial for stabilizing the closed state (Fig. 2, C and D). Our structural analysis of AKT5^D403A^ in the cytosolic C4-symmetric conformation suggests a functional role for Gln407 in interactions associated with the pre-open state (Fig. 3, A to D and fig. S12). Specifically, in the cytosolic C4-symmetric configuration, Gln407 was found to potentially form a hydrogen bond with Asn427 from a neighboring protomer. These observations indicate that Gln407 may participate in the formation of both the closed and pre-open conformations of AKT5.

Various plant growth behaviors, including petiole elongation, rely on turgor-driven cell expansion to optimize leaf blade positioning for efficient light capture (*4*, *48*, *49*). These growth traits are highly dynamic across the day-night cycle and are key determinants of plant competitiveness, influencing whether an individual can extend its leaves above neighboring plants and secure a dominant position within a crowded canopy (*50*). During petiole bending and upward elongation, epidermal cells on the abaxial side elongate more than those on the adaxial side. Although cell-wall remodeling and microtubule rearrangement have been implicated in this process, the primary driving force that promotes cell expansion has remained unknown (*51*, *52*). Since K^+^ is a major osmolyte in plant cells, K^+^ channels represent compelling candidates for generating the turgor pressure required for petiole cell expansion. However, to our knowledge, such K^+^ channels have not been identified. Our data show that AKT5 is involved in petiole elongation (Fig. 6). Considering that AKT5 mediates K^+^ uptake activity (Fig. 4), we propose that AKT5 increases turgor pressure in petiole cells by translocating K^+^ into the cells, resulting in cell elongation. This mechanism differs from K^+^-driven reversible cell-volume control in guard cells and extensor/flexor motor cells in *Samanea saman*, where both inward- and outward-rectifying K^+^ channels enable repeated cycles of expansion and shrinkage (*53*–*58*). Given the irreversible nature of petiole elongation, the inward-rectifying AKT5 channel may function as the primary K^+^ channel in petiole epidermal cells. Consistent with our promoter-reporter and RT-qPCR analyses (Fig. 6, B to D), transcriptome data from the ePlant database confirmed AKT5 expression in rosette leaves and petioles (fig. S14). These data also revealed notably high AKT5 expression in reproductive organs and in early-stage embryos, suggesting that AKT5-mediated K^+^ transport may play additional roles. Studies that identify K^+^ transport systems in the plasma membrane and in organelle membranes, as well as other ion transporters in petiole cells, will help elucidate the molecular mechanisms underlying the precisely tuned growth process.

Despite being a member of the *Shaker* family, AKT5 has long lacked evidence of K^+^ channel activity as well as a defined physiological function; this study fills that long-standing gap. Global food security faces increasing challenges due to a growing population and limited arable land. Plants often grow in dense stands, particularly in agricultural cultures (*6*). Further elucidation of how AKT5 activity is regulated in planta may lead to the development of more efficient high-density cropping systems and ultimately help address future food security challenges.

## MATERIALS AND METHODS

### Protein expression and purification

The codon-optimized coding sequence for *A. thaliana* AKT5 (UniProt: Q9SCX5) was synthesized and subcloned into pFastBac-Dual vector with an N-terminal FLAG tag and a C-terminal 6xHis tag by GenScript (NJ, USA). AKT5^D403A^ was generated using a PCR-based mutagenesis strategy and subcloned into the same vector with the same tags. Baculovirus-infected *Sf9* cells (Thermo Fisher Scientific, MS, USA) were used for overexpression and were cultured in Sf-900 III SFM medium (Thermo Fisher Scientific) at 27°C with shaking at 120 rpm. The P2 virus-transfected cells were harvested approximately 60 hours post-infection.

Cell pellet obtained from 500 ml for AKT5 or 1.5 L for AKT5^D403A^ cultures were resuspended in extraction buffer (100 ml for AKT5, 150 ml for AKT5^D403A^) containing 10 mM lauryl maltose neopentyl glycol (LMNG), 2 mM cholesteryl hemisuccinate (CHS), 300 mM KCl, 20 mM Tris-HCl (pH 8.0) and a protease inhibitor cocktail (cOmplete; Roche, Basel, Switzerland). Membranes were solubilized by rotation at 4°C for 1.5 hours. Insoluble materials were removed by centrifugation at 20,000g for 30 min at 4°C. The supernatant was added to 2 ml of anti-Flag M2 affinity resin (Sigma-Aldrich, MO, USA) and incubated for 1 hour at 4°C with gentle rotation. The resin was rinsed with 20 column volumes (CV) of wash buffer (0.02% GDN, 300 mM KCl, and 20 mM Tris-HCl pH 8.0). Bound proteins were eluted with 8 CV of wash buffer supplemented with 200 μg/ml 3 × FLAG peptide. Eluted proteins were concentrated to approximately 550 μL using an Amicon Ultra centrifugal filter (100 kDa MWCO; Merck, Darmstadt, Germany) and further purified by size-exclusion chromatography on a Superose 6 Increase column equilibrated with SEC buffer (0.02% GDN, 150 mM KCl, 20 mM Tris-HCl pH 8.0 and 2 mM DTT).

### Single-particle cryo-EM data acquisition

Purified AKT5 protein (3 μl; 2 mg/ml for the wild-type AKT5 and 4 mg/ml for AKT5^D403A^) was applied to freshly plasma-cleaned holey carbon grids (Quantifoil, R1.2/1.3, 200 mesh, Cu). The grids were blotted for 1.5 s (AKT5) or 2.0 s (AKT5^D403A^) at 100% humidity and 4°C using Vitrobot Mark IV (Thermo Fisher Scientific), and plunge-frozen in liquid ethane cooled by liquid nitrogen. The vitrified grids were stored in liquid nitrogen until data collection. Grids were transferred to CRYO ARM 300 II (JEOL, Tokyo, Japan) operated at 300 kV. Micrograph movies were collected at 60,000 × nominal magnification, corresponding to a physical pixel size of 0.788 Å for AKT5, 0.806 Å for AKT5^D403A^), with a total dose of 60 electrons per Å^2^ using a K3 Camera direct electron detector (Gatan) controlled by the SerialEM program (*59*) and used for subsequent data processing.

### Image processing

For the wild-type AKT5, all data processing on a total of 7,950 micrographs was performed using CryoSPARC49. Briefly, motion correction and CTF estimation were carried out using Patch Motion Correction and Patch CTF Estimation, respectively. Manually Curate Exposures was used to eliminate disqualified micrographs determined by thresholding and manual selection. Particle picking was performed on the selected micrographs using Template Picker. Extracted particles were applied to 2D classifica-tions, and a total of 54,571 particles were used for Non-Uniform Refinement with C2 symmetry, yielding a final consensus reconstruction at an overall resolution of 3.62 Å. For AKT5D403A, image processing was performed in CryoSPARC using 4,600 micro-graphs following the same workflow as for the wild-type protein. After 2D classifications, 465,795 particles were selected for Ab-Initio Reconstruction. Subsequent 3D Classification into 12 classes revealed two distinct particle populations. One population, comprising 186,912 particles, was refined by Non-Uniform Refinement C4 symmetry to a resolution of 2.60 Å, while the second population, containing 51,494 particles, yielded a reconstruction of 3.16 Å resolution.

### Model building

A provisional model of AKT5 was created by SWISS-MODEL (*60*) using the structure of AKT1 (PDB: 7WSW) as a template. For AKT5^D403A^, AKT1^D379A^ (PDB: 7FCV) (*37*) was used as a template to generate a provisional model. The provisional models were roughly fitted to the reconstructed cryo-EM maps using UCSF ChimeraX (*61*). During model building, the chemical properties of amino acids were taken into consideration, and all residues were manually inspected. Iterative cycles of manual rebuilding were performed in Coot software (*62*), following real-space refinement with the use of Phenix (*63*).

Due to the absence of interpretable density, N-terminal residues preceding approximately position 70 and the C-terminal residues following approximately position 520 were omitted from the final models. All molecular graphics in this study were prepared using PyMOL (version 3.1.1, Schrödinger, LLC.), UCSF ChimeraX, VMD (University of Illinois at Urbana-Champaign) and BIOVIA Discovery Studio 2021 (Dassault Systèmes).

### Structural modeling of AKT5 in C2/C4 symmetry for morphing movie

To visualize changes in CNBHD interactions between C2 and C4 symmetries, C2- and C4- symmetric structural models of AKT5 were generated and used to produce a morphing movie. To fill in the missing regions, the C2- and C4-symmetric structures of AKT1 (PDB: 7WSW and 7FCV) (*37*) were used as templates to build corresponding C2- and C4- symmetric AKT5 models using SWISS-MODEL. These models were then used to replace the missing regions in the experimentally determined C2 (WT, 9X0B) and C4 (D403A, 9X0C) AKT5 structures obtained in this study. Using SWISS-MODEL, final model structures encompassing the same residue range (I70–L533) were generated for both symmetries. A morphing movie between the resulting C2 and C4 AKT5 models was generated using VMD.

### Growth complementation assay

The coding sequences of CBL1–10 were subcloned into the pYGPE15 vector, CIPK1–23 into the p414GPD vector, and K^+^ channel genes, including AKT5, into YCplac111. Yeast transformation was performed using a commercial kit (*S. cerevisiae* Direct Transformation Kit, FUJIFILM Wako Pure Chemical Corporation, Osaka, Japan). For screening of CBLs-CIPKs activating AKT5, transformed strains were inoculated into 50–60 μl of arginine phosphate medium (8 mM phosphoric acid, 2 mM MgSO_4_, 0.2 mM CaCl_2_, 20 μg/ml adenine, 20 μg/ml histidine, 40 μg/ml leucine, 20 μg/ml uracil, 30 μg/ml tryptophan, 2% glucose, 500 μg/ml boric acid, 40 μg/ml CuSO_4_, 100 μg/ml KI, 500 μg/ml FeSO_4_, 400 μg/ml MnSO_4_, 900 μg/ml ammonium molybdate, 400 μg/ml ZnSO_4_, 400 μg/ml nicotinic acid, 400 μg/ml pyridoxine, 400 μg/ml thiamine, 400 μg/ml pantothenate, 200 μg/ml biotin, pH was adjusted to 6.5 with L-arginine; used as -Ura-Trp or -Ura-Trp-Leu) and incubated at 30°C for 20–24 hours. Subsequently, 5 μl of the culture diluted to an OD_600_ of 0.1 was spotted onto arginine phosphate agar medium (-Ura-Trp or -Ura-Trp-Leu) and incubated at 30°C.

For detailed growth complementation assays, strains were cultured in 3 ml synthetic complete (SC) medium and cultured for 20–24 hours, washed three times with K^+^-free arginine phosphate medium, and diluted to OD_600_ = 0.1. Five microliters of the 10^1^–10^4^ serial dilutions were spotted onto arginine phosphate agar plates and incubated at 30°C for 3 days.

### Electrophysiological experiments using *X. laevis* oocytes

For expression in *X. laevis* oocytes, cDNAs encoding K^+^ channel genes and CIPK10K^41N^ were subcloned into a pYES2-derived vector, pOMU1 (Invitrogen, Carlsbad, CA, USA) (*64*). AKT5 variants were generated using the In-Fusion HD cloning kit (Takara Bio, Shiga, Japan) as described previously. (*55*) CBL1/9 and CIPK10/23 were subcloned into pGEMKN vectors. cRNAs were synthesized from linearized plasmids using the mMESSAGE mMACHINE T7 Transcription Kit (Ambion, TX, USA). Two-electrode voltage clamp recordings were performed while perfusing oocytes with a solution composed of 120 mM KCl, 1 mM MgCl_2_, 1 mM CaCl_2_, 1 mM LaCl_3_, and 10 mM Hepes-NaOH (pH7.3) (*65*). Volage steps from +10 mV to −170 or −190 mV (−20 mV increments, 500 ms duration) were applied from a holding potential of −40 mV. Recordings and data analysis were performed using an AxoClamp 2B amplifier (Molecular Devices, CA, USA) and an Axon Digidata 1550 System (Molecular Devices, CA, USA).

### Electrophysiological experiments using COS-7 cells

The DNA encoding AKT5, AKT6, CBL9, and CIPK10 were subcloned into the pcDNA3 vector. COS-7 cells were transfected with those clones and a transfection indicator (yellow fluorescent protein, YFP). 24–48 h after transfection, the macroscopic current was recorded from cells expressing YFP, using whole cell patch clamp technique with an Axopatch 200B amplifier, Digidata 1550B and pClamp 11 software (Molecular Devices, CA, USA), as previously described (*66*). The bath solution for the current recording was composed of 180 mM mannitol, 50 mM KCl, 1 mM CaCl_2_, 3 mM MgCl_2_, 10 mM Hepes (pH 7.4 adjusted with KOH). The pipette solution contained 130 mM KCl, 5 mM Na_2_-ATP, 3 mM EGTA, 0.1 mM CaCl_2_, 10 mM Hepes, 4 mM MgCl_2_, 0.3 mM GTP (pH 7.3 adjusted with KOH). Once the whole cell configuration was established, the cell was held at −20 mV. Test pulses (−200 mV to 0 mV, 20 mV increment, for 400 ms) were applied every 7 sec, and the P/8 protocol was performed to exclude the leak current component. Steady-state current amplitudes were calculated as the average of the current amplitude at 362–392 ms and normalized by membrane capacitance to obtain current density.

### Mass spectrometry analysis of AKT5

AKT5 protein was expressed in baculovirus-infected *Sf9* cells as described above, purified by gel filtration, and concentrated to approximately 1 mg/ml. 8xHis-CBL9 or 8xHis-CIPK10 was subcloned into the pTD1 vector, and mRNA was synthesized using the RiboMAX Large Scale RNA Production System-T7 (Promega, WI, USA). Proteins were synthesized in a silkworm cell-free system as described previously (Silk Renaissance Co., Ltd.) (*47*). In vitro phosphorylation and trypsin digestion were essentially carried out as described (*67*). AKT5, CBL9 and CIPK10 were incubated in 50 μL reaction buffer [50 mM Tris-HCl (pH 7.5), 5 mM MgCl_2_, 5 mM MnCl_2_, 50 μM ATP, 1% (v/v) protease inhibitor cocktail (Sigma-Aldrich)] at 30°C for 30 min. Reactions were terminated with trichloroacetic acid. Protein pellets were resuspended in 50 mM ammonium bicarbonate and incubated in the presence of 10 mM DTT for 30 min at 24°C, followed by alkylation with 40 mM iodoacetamide for 30 min at 24°C in the dark, and digested overnight at 37°C with 0.2 μg trypsin (Promega). Peptides were desalted using StageTips made in-house with C18 Empore disks (GL Sciences), dried, resuspended in 20 μL of 2% (v/v) acetonitrile, 0.1% (v/v) formic acid, and analyzed on an Easy-nLC 1200 (Thermo Fisher Scientific) coupled to an Exploris 480 quadrupole-Orbitrap mass spectrometer (Thermo Fisher Scientific) equipped with FAIMS Pro high-field asymmetric waveform aerodynamic ion mobility spectrometry (Thermo Fisher Scientific). Peptide/protein identification and MS1-based label-free quantification were carried out using Proteome Discoverer 2.5 (Thermo Fisher Scientific).

### Plasmids and *Arabidopsis* transformation

To generate a construct expressing the GUS reporter gene under the control of the *AKT5* promoter (*P_AKT5_*-*GUS*), an *AKT5* promoter fragment (3 kb) was amplified by PCR from *Arabidopsis* genome using the following primer pair:, 5′-TGATTACGCCAAGCTTATGTAATGGACCAATGCT-3′ (*Hind*III site underlined) and 5′-AGGGACTGACCACCCGGGACCGATCGATGAATTTGA-3′ (*Sma*I site underlined). The resultant *AKT5* promoter fragment was subcloned into the *Hind*III-*Smal*I sites of the binary vector containing the *GUS* gene (*68*) using In-Fusion cloning kit (TAKARA Japan). To construct a GFP-fused AKT5 expression cassette driven by *AKT5* promoter (*P_AKT5_-GFP*-*AKT5*), the GFP coding sequence was amplified by PCR using the primers 5′-TCATCGATCGGTCCCATGGTGAGCAAGGGCGAG-3′ and 5′-CAATACCCATTCCTCCTCCTCCCTTGTA-3′, and *AKT5* sequence amplified by PCR using a set of primers 5′-GAGGAGGAGGAATGGGTATTGAGAAGAGG-3′ and 5′-GATCGGGGAAATTCGAGCTCTCAAGAAACCTTAAGAAG-3′. The amplified GFP and AKT5 fragments were subsequently inserted into the *Sma*I and *Sac*I sites of the above binary vector for expression *P_AKT5_*-*GUS* using In-Fusion cloning kit (TAKARA Japan). To construct a GFP-fused AKT5 expression cassette driven by CaMV 35S promoter (*P_35S_-GFP*-*AKT5*), the GFP-fused AKT5 coding sequence was amplified by PCR using the plasmid (*PAKT5-GFP-AKT5*) as a template and the primers 5′-CTAGAGGATCCCCATGGTGAGCAAGGGCGAG-3′ and 5′-CAATACCCATTCCTCCTCCTCCCTTGTA-3′ and 5′-GATCGGGGAAATTCGAGCTCTCAAGAAACCTTAAGAAG-3′. The amplified GFP and AKT5 fragments were subsequently inserted into the *Sma*I and *Sac*I sites of the binary vector using In-Fusion cloning kit (TAKARA Japan). *A. thaliana* was grown in soil or on 1/2 MS agar plates in growth chambers under a 16-hour light and 8-hour dark photoperiod at 22°C. Seeds of the *akt5* mutant (SALK_015179C) were obtained from the *Arabidopsis* Biological Resource Center (https://abrc.osu.edu/). Transgenic lines including *P_AKT5_*-*GUS/*WT, *P_AKT5_*-*GFP*-*AKT5*/*akt5*, and *P_35S_-GFP*-*AKT5*/WT, were generated by the floral dip method (*69*) using *Agrobacterium tumefaciens* GV3101 strain as described previously (*68*).

### GUS reporter assay of *Arabidopsis*

GUS staining was performed as previously described (*68*, *70*). *P_AKT5_*-*GUS* plants were fixed in 90% acetone for 15 min on ice, rinsed with 50 mM phosphate buffer (pH 7.0), and incubated in GUS staining solution containing 1.9 mM 5-bromo-4-chloro-3-indolyl-β-D-glucuronide (X-Gluc), 3 mM K_4_Fe(CN)_6_, 0.5 mM K_3_Fe(CN)_6_, 20% (v/v) MeOH, and 50 mM phosphate buffer (pH 7.0). After staining, samples were destained in 70% ethanol, dehydrated through an ethanol series, and embedded in Technovit 7100 (Heraeus Kulzer, Tokyo, Japan). Sections (6 μm) were prepared using a Leica RM2145 microtome (Leica, Wetzlar, Germany). The sections were stained with 0.01% neutral red (FUJIFILM Wako Pure Chemical Corporation, Osaka, Japan) for 1 min, rinsed twice with water, sealed, and photographed.

### RT-qPCR analysis

Plants were germinated and grown on 1/2 MS medium for one week. Total RNA was extracted using TRI Reagent (Molecular Research Center Inc., OH, USA). cDNA was synthesized using ReverTra Ace qPCR RT Master Mix with gDNA Remover (TOYOBO Co. Ltd., Osaka, Japan) by sequential incubation at 37°C for 15 min, 50°C for 5 min, and 98°C for 5 min. RT-qPCR was performed using a QuantStudio3 (Thermo Fisher Scientific, MS, USA) with the KAPA SYBR FAST qPCR Master Mix (2×) Kit (KAPA Biosystems, MA, USA). The thermal cycling conditions were as follows: initial denaturation at 95°C for 3 min, followed by 40 cycles of 95°C for 3 s and 60°C for 20 s. The polyubiquitin gene *UBQ10* (At4g05320) was used as an internal reference. The primers sequences were as follows: 5′-CCCTAACGGGAAAGACGATTAC-3′ (forward) and 5′-AGAGTTCTGCCATCCTCCAAC-3′ (reverse) for *UBQ10*; 5′-AAAATACGGAGGAGATATCTCACTC-3′ (forward) and 5′-CTTATTCCAGTCCTGGTAGATGC-3′ (reverse) for *AKT5*.

### Measurement of root length of *Arabidopsis* seedlings

*A. thaliana* seedlings were grown on 1/2 MS agar plate for 9 days under a 16-hour light (56–80 μmol m^−2^ s^−1^) and 8-hour dark photoperiod at 22°C. Seedlings were photographed on the plates, and root length was measured using ImageJ software (*71*).

### Petiole length and angle measurement

For petiole length and angle measurements, *A. thaliana* was grown in soil in growth chambers with 16 hours of light (75 μmol m^−2^ s^−1^ from Valoya BX120 NS1 + FR) and 8 hours of dark at 21°C. At 15 days after sowing, plants were transferred to an imaging setup and monitored for 24 hours. The imaging setup was implemented as described previously (*48*). Briefly, plants were maintained under their native light-dark regime, and IR illumination (peak wavelength, 950 nm) was added to enable imaging during the dark phase. Raspberry Pi mini-computers equipped with IR camera were positioned in front of the plants, with one focal leaf per plant aligned parallel to the camera. Each camera imaged two plants, and nine cameras operated simultaneously, all taking a picture every minute.

Using an automated image extraction and analysis pipeline (https://github.com/Pierik-Lab/RPi-Image-analysis-workflow/), petiole lengths and angles relative to the horizontal were calculated and averaged over the 14–16 biological replicates. The data were plotted against Zeitgeber time for each genotype. Separate time-lapse video’s were made to illustrate leaf movement. Approximately 12-day-old seedlings were photographed with a digital camera (SIONYX AURORA Pro; SIONYX, MA, USA) in a growth chamber under 16 hour light (70 μmol m^−2^ s^−1^) and 8 hour dark photoperiod at 22°C. During the dark phase, plants were illuminated with dim green light (0.03 μmol m^−2^ s^−1^) to enable image acquisition without perturbing dark conditions.

### Growth under crowded conditions and fresh-weight measurement of *Arabidopsis*

*A. thaliana* (Col-0) and the *akt5* mutants were grown under crowded conditions in regularly spaced 4-ml or 15-ml tubes placed in tube racks. The bottoms of the tubes were removed and covered with mesh netting to prevent soil loss, and soil was packed into the tubes. One plant was grown per tube, and crowding was achieved by placing the tubes in close proximity to each other. The plants were then grown in a growth chamber under a 16 hour light period (80 μmol m^−2^ s^−1^) and an 8 hour dark period at 22°C with 85% relative humidity. At 23 days after sowing, shoots were harvested into 5-ml tubes, and the fresh weight was measured immediately.

### Fluorescence observation of *Arabidopsis*

Root tissues from 2-week-old *P_35S_-GFP*-*AKT5* plants grown on solid 1/2 MS medium supplemented with hygromycin (2 μg/ml) were harvested and prepared for analysis. Fluorescence observations were performed using a microscope (IX-83; Olympus, Tokyo, Japan). GFP fluorescence was excited at 488 nm.

### Sequence and phylogenetic analysis

Multiple protein sequence alignments and phylogenetic tree reconstructions were performed using GENETIX (*72*) and Geneious Prime 2019 software (http://www.geneious.com/).

### Statistical analysis

Statistical analyses were performed using Excel (Microsoft Corp., WA, USA) or GraphPad Prism version 9.5.1 for Windows (GraphPad Software, MA, USA, www.graphpad.com). The exact value of n, definition of error bars, and levels of statistical significance are included in the figures, and figure legends. Asterisks denote statistical significance for the data statistically analyzed using a two-tailed Student’s *t* test (*P < 0.05). For data statistically analyzed by ANOVA, different letters represent statistical significances (*P* < 0.05).
