# Supplementary material for: AKT5 is a bona fide potassium channel and controls petiole growth in Arabidopsis
Source: Sci Adv. 2026 Sep 2;12(36):eaeh7630. doi: 10.1126/sciadv.aeh7630 (PMC13537238; doi:10.1126/sciadv.aeh7630)
Supplement: Supplementary file 1 [file sciadv.aeh7630_sm.pdf]

Science Advances
AAAS

Supplementary Materials for

AKT5 is a bona fide potassium channel and controls petiole growth in *Arabidopsis*

Yuki Muraoka *et al.*

Corresponding author: Nobuyuki Uozumi, uozumi@tohoku.ac.jp

Sci. Adv. **12**, eaeh7630 (2026)
DOI: 10.1126/sciadv.aeh7630

The PDF file includes:

Figs. S1 to S14
Table S1
Legends for movies S1 and S2

Other Supplementary Material for this manuscript includes the following:

Movies S1 and S2

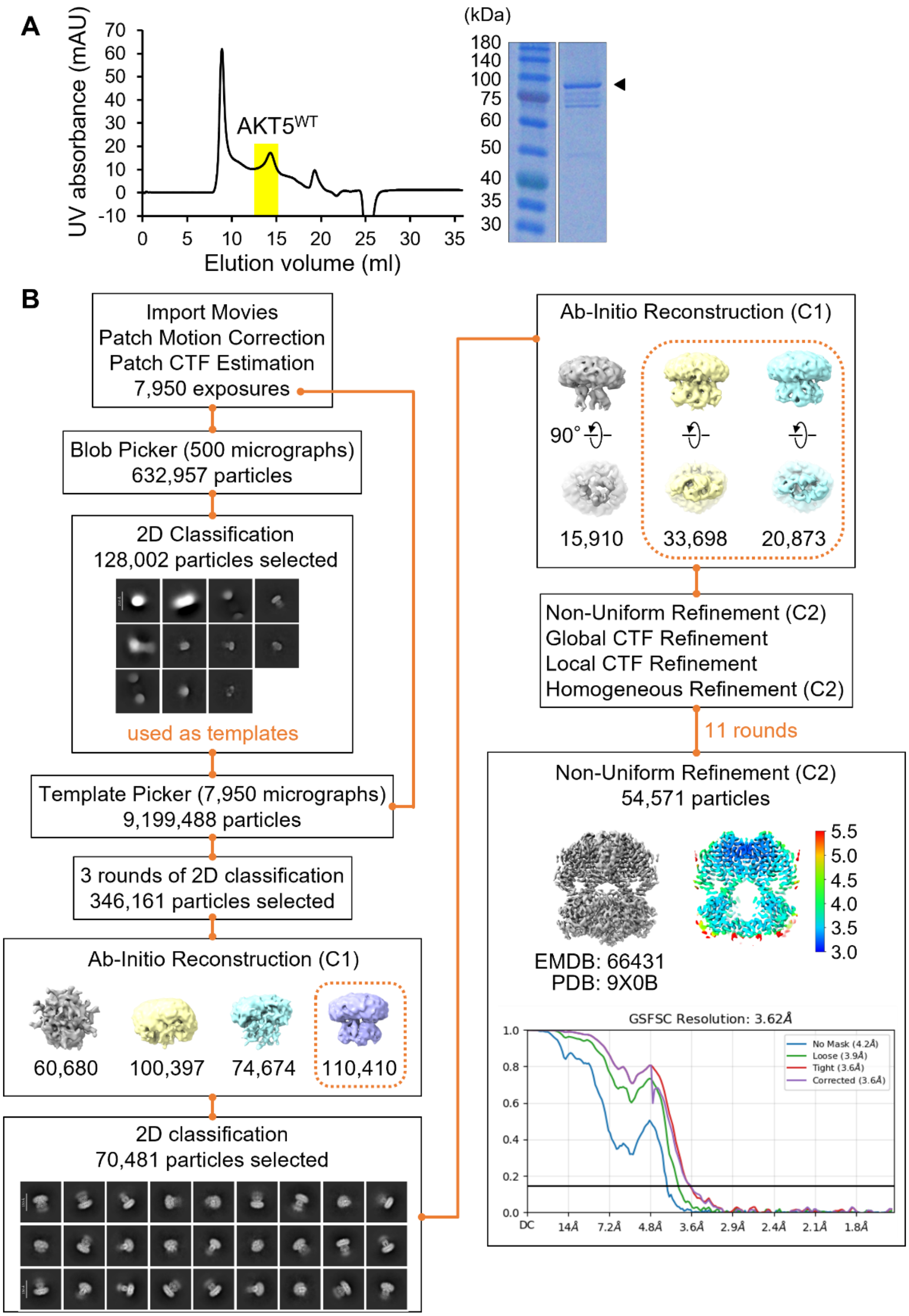

Fig. S1. Cryo-EM analysis of AKT5.

(**A**) Representative size-exclusion chromatograms and Coomassie Brilliant Blue-stained SDS-PAGE gels. Fractions highlighted in yellow were collected and used for cryo-EM grid freezing preparation. (**B**) Cryo-EM data processing pipeline.

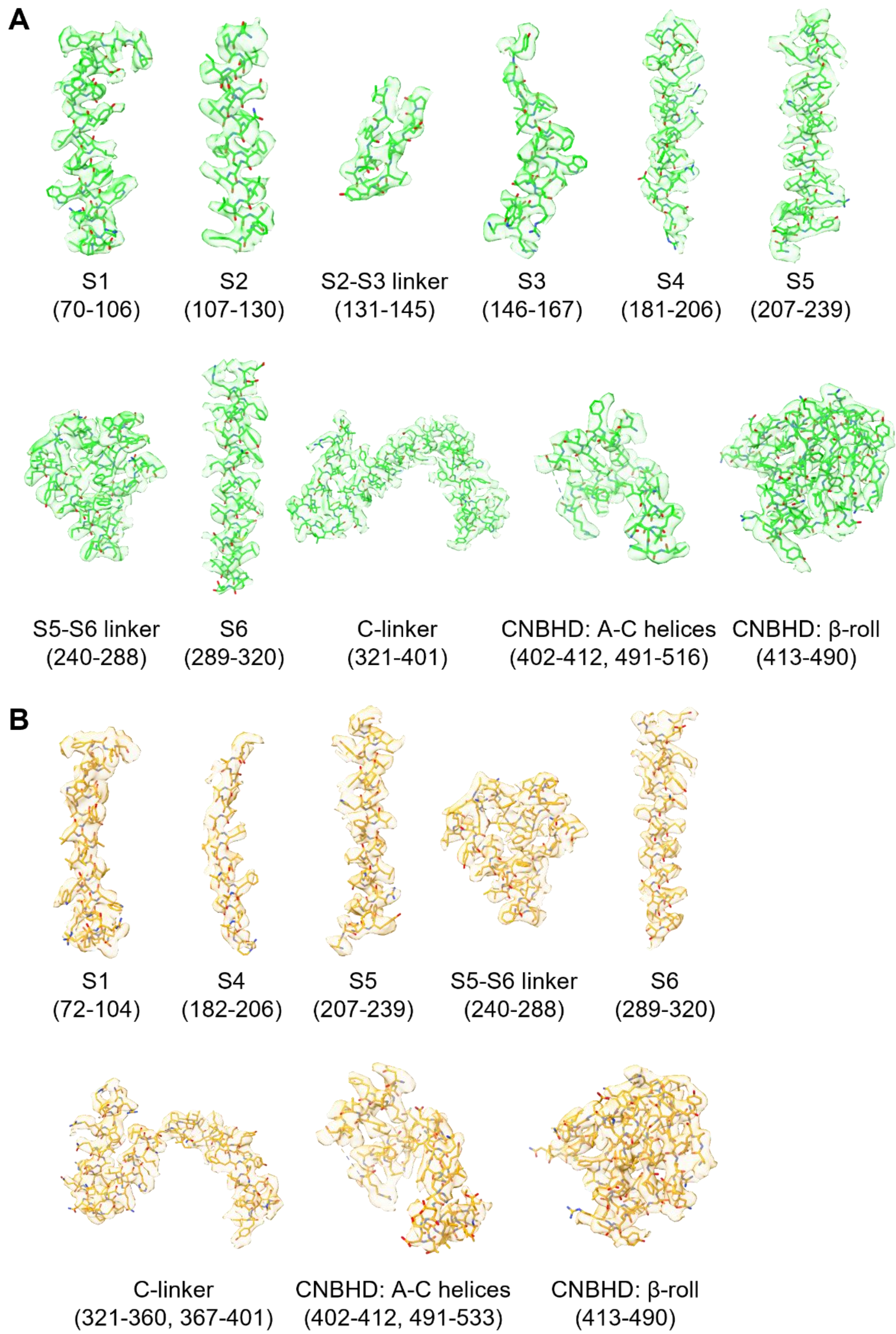

Fig. S2. Cryo-EM density of AKT5.

Cryo-EM density maps and corresponding structural models of Mol I/I’ (**A**) and Mol II/II’ (**B**) for the indicated regions.

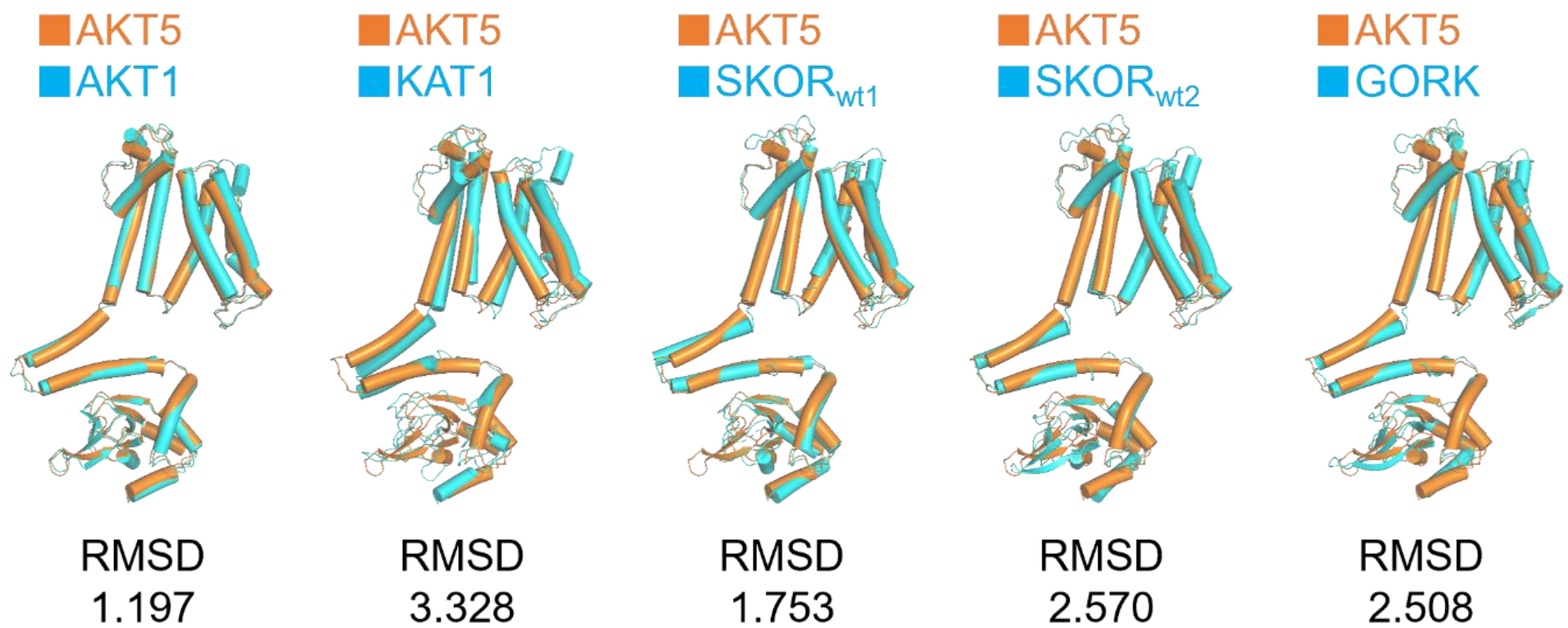

Fig. S3. The structural features of AKT5.

The RMSD (Root Mean Square Deviation) values were calculated using PyMOL based on the structures of AKT5, AKT1 (PDB: 7WSW), KAT1 (PDB: 7CAL), SKOR$_{wt1}$ (PDB: 8JET), SKOR$_{wt2}$ (PDB: 8JEU), and GORK (PDB: 9J0X).

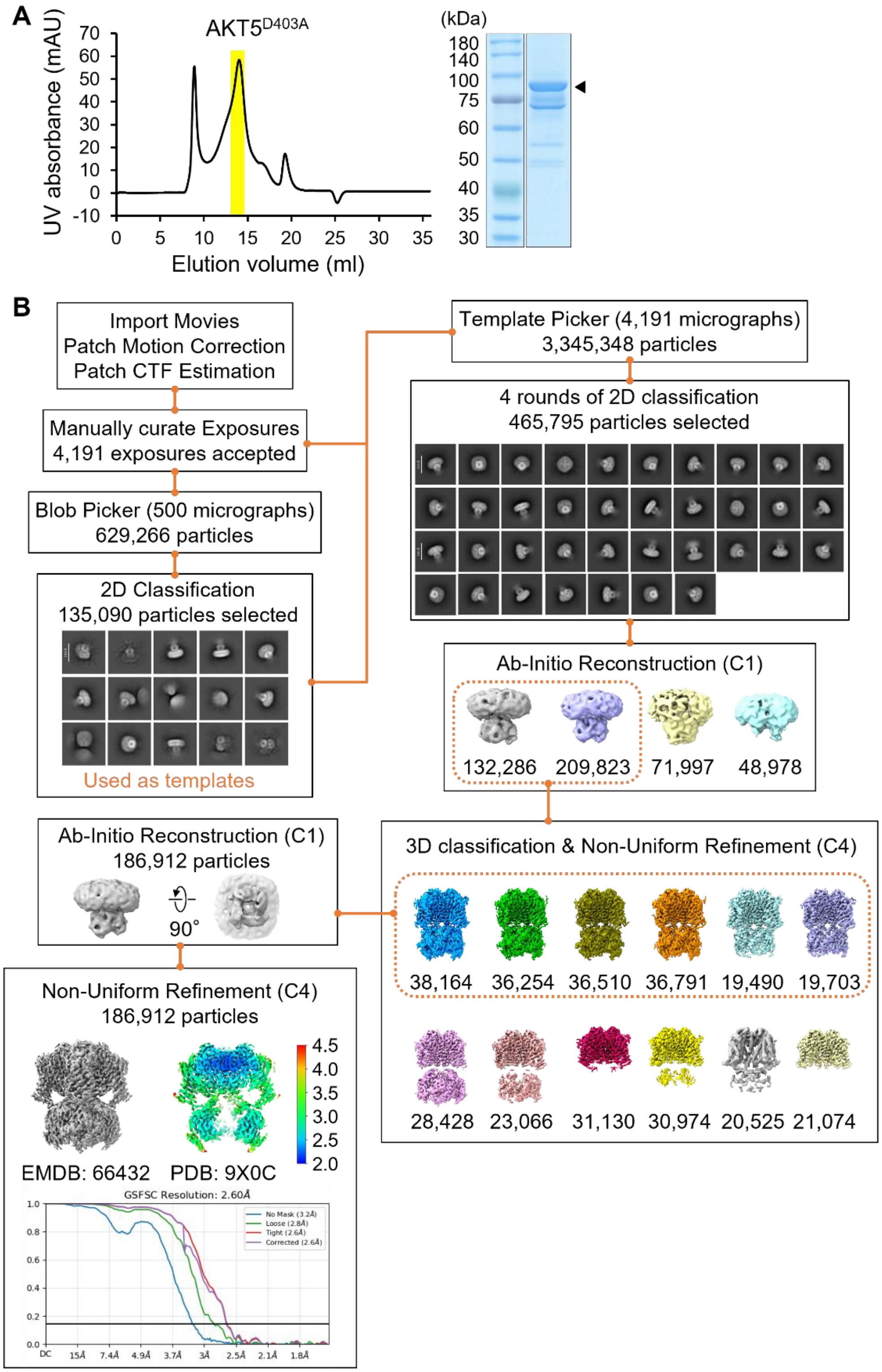

Fig. S4. Cryo-EM analysis of AKT5^{D403A}.

(**A**) Representative size-exclusion chromatography profiles and Coomassie Brilliant Blue-stained SDS-PAGE gels. Fractions highlighted in yellow were collected and used for grid freezing preparation. (**B**) Cryo-EM data processing pipeline.

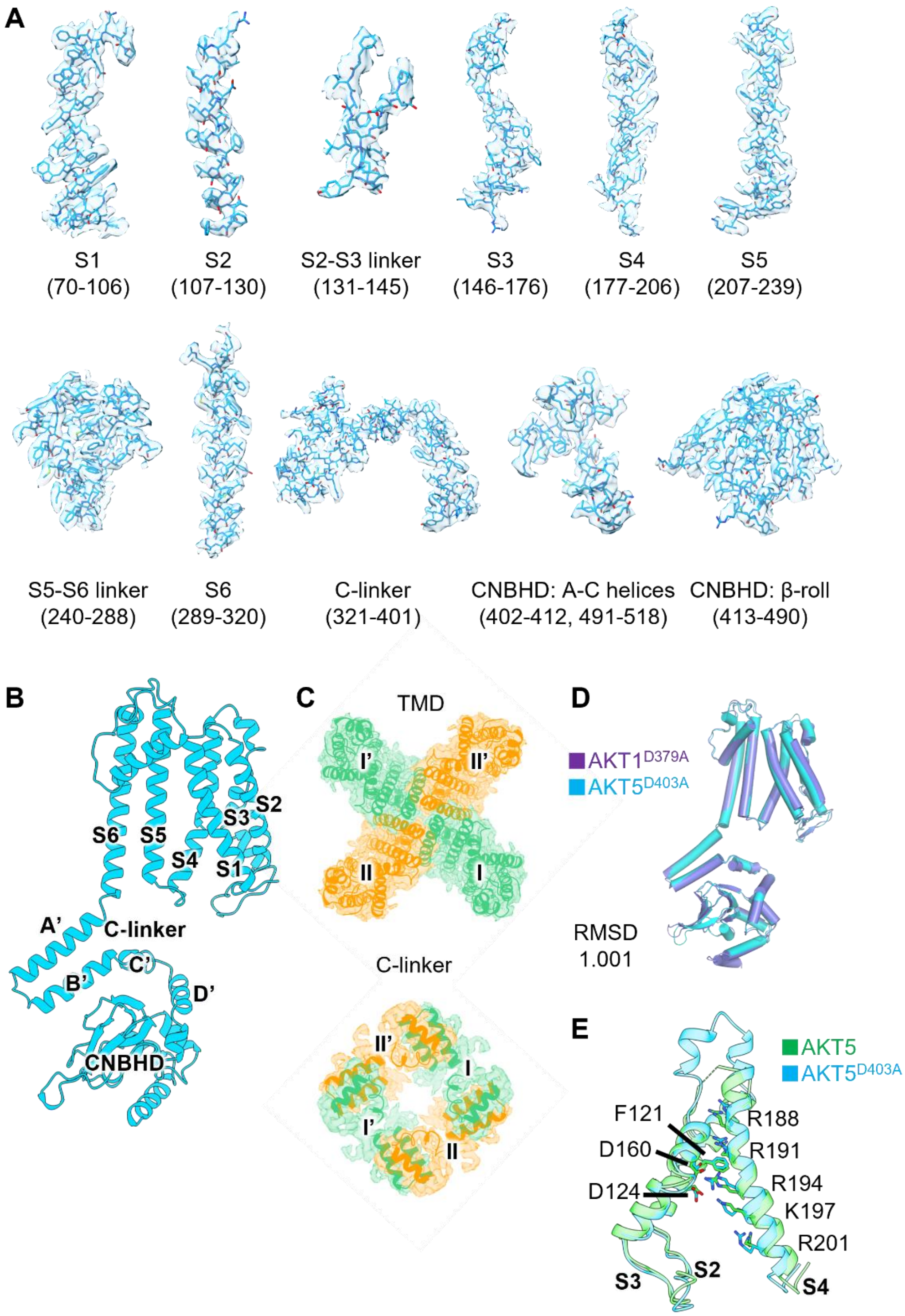

Fig. S5. The structural features of AKT5^{D403A}.

(**A**) Cryo-EM structures and corresponding density maps of AKT5^{D403A} for the indicated regions. (**B**) Overall view of a protomer of AKT5^{D403A}, with representative domains labeled. (**C**) Domain layers viewed sectionally from the intracellular side. (**D**) RMSD values were calculated using PyMOL based on the structures of AKT5^{D403A} and AKT1^{D379A} (PDB: 7FCV). (**E**) Side view of the VSD from AKT5 (Mol I/I') and AKT5^{D403A}.

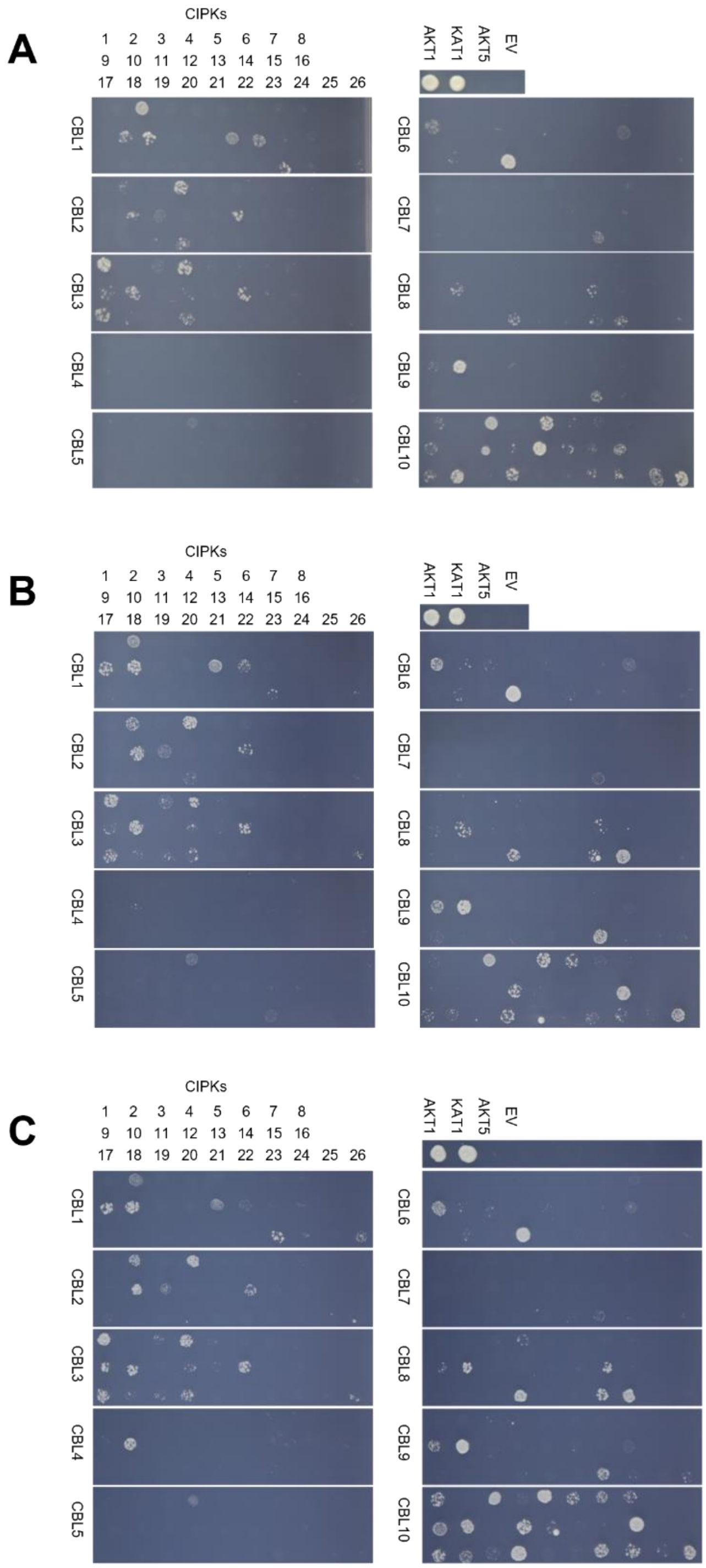

Fig. S6. Screening of CBL-CIPK pairs that activate AKT5 in yeast.

(**A**-**C**) Growth complementation assays were performed in *S. cerevisiae* 9.3 strain on agar medium supplemented with 0.5 mM K^+. Triplicate assays were conducted using 247 combinations of AKT5 with CBL1-10 and CIPK1-26 to identify CBL-CIPK pairs that activate AKT5 channel activity.

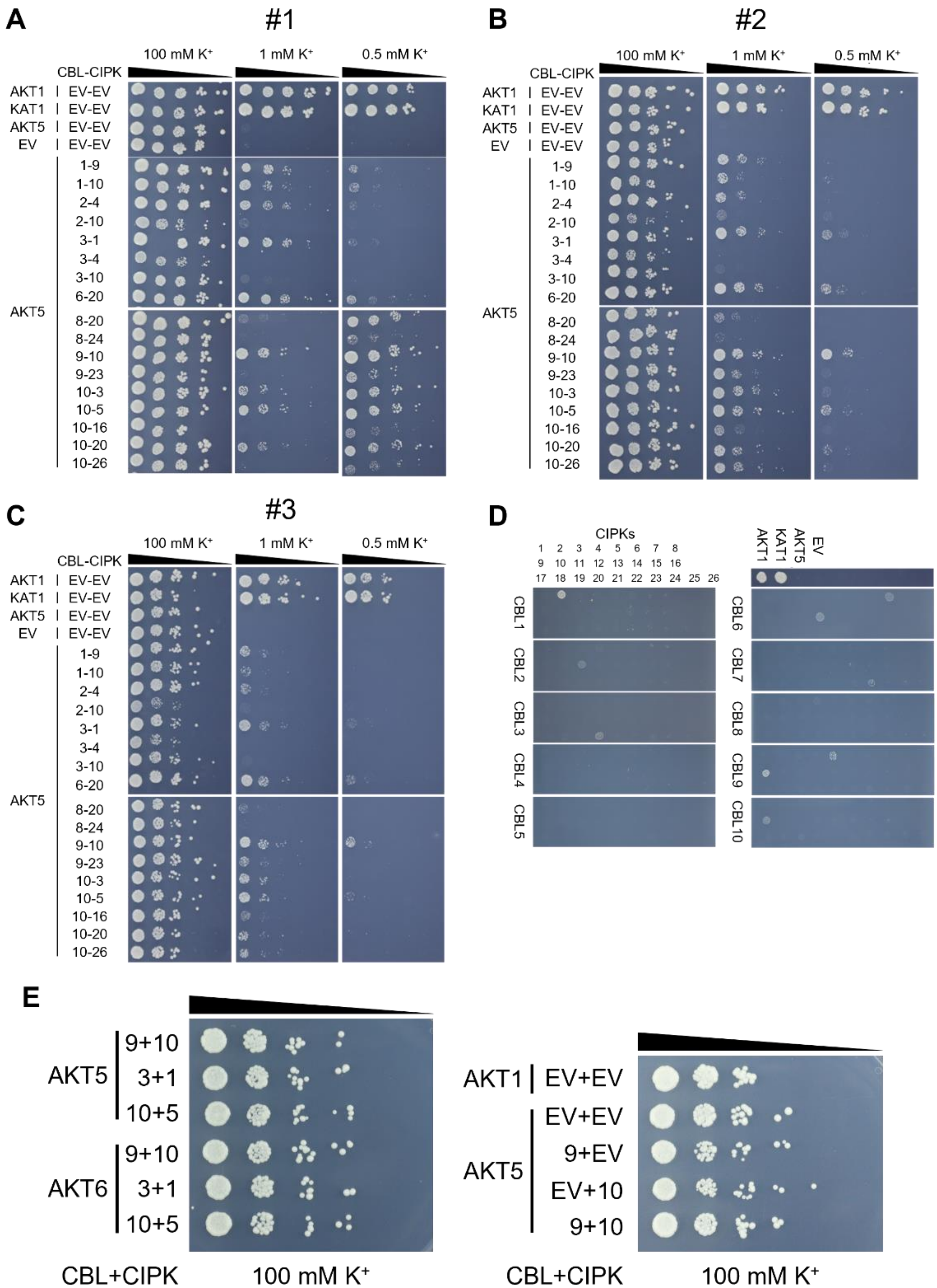

A
#1
100 mM K⁺
1 mM K⁺
0.5 mM K⁺
CBL-CIPK
AKT1 | EV-EV
KAT1 | EV-EV
AKT5 | EV-EV
EV | EV-EV
AKT5
1-9
1-10
2-4
2-10
3-1
3-4
3-10
6-20
8-20
8-24
9-10
9-23
10-3
10-5
10-16
10-20
10-26
B
#2
C
#3
D
CIPKs
1 2 3 4 5 6 7 8
9 10 11 12 13 14 15 16
17 18 19 20 21 22 23 24 25 26
CBL1
CBL2
CBL3
CBL4
CBL5
AKT1 KAT1 AKT5 EV
CBL6
CBL7
CBL8
CBL9
CBL10
E
AKT5
9+10
3+1
10+5
AKT6
9+10
3+1
10+5
CBL+CIPK
100 mM K⁺
AKT1 | EV+EV
AKT5
EV+EV
9+EV
EV+10
9+10
CBL+CIPK
100 mM K⁺

Fig. S7. Screening of selected CBL1-10 and CIPK1-26 pairs that activate AKT5 activity in a yeast screening assay on agar medium supplemented with 0.5 mM K^+.

(**A-C**) Triplicated growth tests (#1-3) of selected CBL1-10 and CIPK1-26 pairs, based on the results of Supplemental Figure S6. (**D**) The growth test of *S. cerevisiae* strain 9.3 expressing CBL1-10 and CIPK1-26 pairs in the absence of AKT5 on agar supplemented with 0.5 mM K^+. The following nine CBL–CIPK combinations caused auto-activation in the absence of AKT5: CBL1–CIPK2, CBL2–CIPK11, CBL3–CIPK20, CBL6–CIPK16, CBL6–CIPK20, CBL7–CIPK23, CBL9–CIPK5, CBL9–CIPK9, and CBL10–CIPK9. (**E**) Dilution series of yeast strain 9.3 transformed with AKT1, AKT5, and AKT6 together with one of three CBL-CIPK pairs, CBL9-EV, EV-CIPK10 or EV-EV, grown on agar plates supplemented with 100 mM K^+.

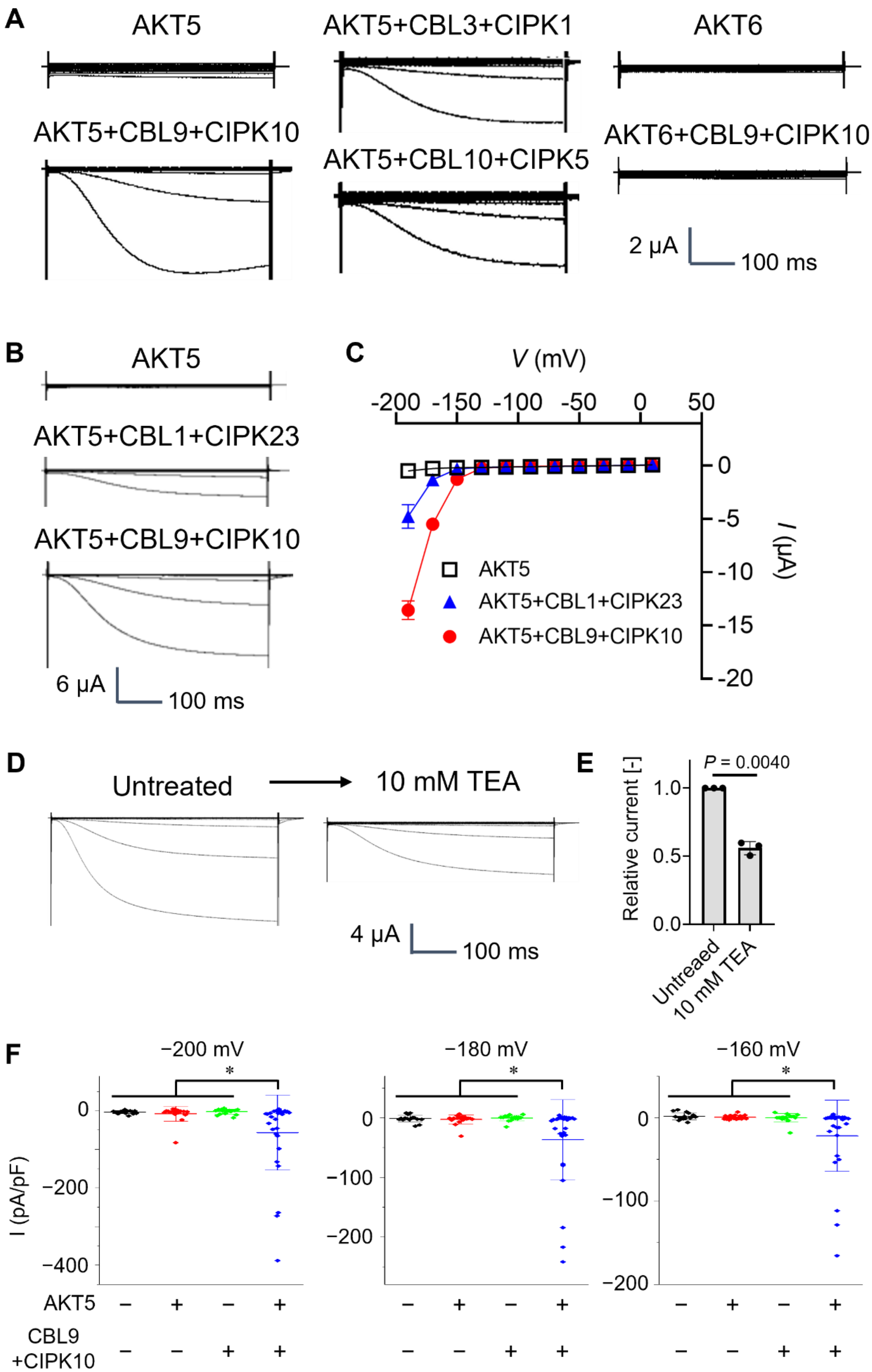

A
AKT5
AKT5+CBL3+CIPK1
AKT6
AKT5+CBL9+CIPK10
AKT5+CBL10+CIPK5
AKT6+CBL9+CIPK10
2 μA
100 ms
B
AKT5
AKT5+CBL1+CIPK23
AKT5+CBL9+CIPK10
6 μA
100 ms
C
V (mV)
-200 -150 -100 -50 0 50
0 -5 -10 -15 -20
I (μA)
AKT5
AKT5+CBL1+CIPK23
AKT5+CBL9+CIPK10
D
Untreated
10 mM TEA
4 μA
100 ms
E
P = 0.0040
Relative current [-]
1.0 0.5 0.0
Untreaed
10 mM TEA
F
−200 mV
−180 mV
−160 mV
I (pA/pF)
AKT5
CBL9
+CIPK10

Fig. S8. AKT5 is activated by CBL-CIPK complexes to function as an inward-rectifying K^+ channel in two heterologous expression systems.

(**A**) Representative current profiles recorded from *Xenopus laevis* oocytes. CBL3-CIPK1 and CBL10-CIPK5 activated AKT5 similarly to CBL9-CIPK10, whereas CBL9-CIPK10 did not activate AKT6. Currents for AKT5-expressing oocytes were elicited by voltage steps from +10 to −170 mV (Δ = −20 mV), and those for AKT6-expressing oocytes from +10 to −190 mV (Δ = −20 mV). Currents were recorded in the solution containing 120 mM K^+. Images were cropped to display only the relevant important regions. (**B**) Representative current traces recorded from *Xenopus laevis* oocytes expressing AKT5 alone, AKT5+CBL9+CIPK10, or AKT5+CBL1+CIPK23. Currents were elicited by voltage steps from +10 to −190 mV (Δ = −20 mV). Currents were recorded in the solution containing 120 mM K^+. Images were cropped to display only the relevant important regions. (**C**) *I-V* relationship of steady-state currents in Xenopus oocytes versus applied voltages in (B). The data are presented as mean ± SD (n = 6). (**D**) Representative current traces recorded from Xenopus oocytes expressing AKT5+CBL9+CIPK10 before (Untreated) and after application of 10 mM TEA. Currents were elicited by voltage steps from +10 to −190 mV (Δ = −20 mV). Images were cropped to display only the relevant important regions. (**E**) Relative current amplitudes of AKT5+CBL9+CIPK10-expressing oocytes at −190 mV before and after application of 10 mM TEA (mean ± SD, n = 3). Statistical significance was assessed using a paired two-tailed *t*-test. (**F**) Steady-state current densities measured at −200, −180, −160 mV (mean ± SD) in COS-7 cells expressing YFP (n = 17), YFP+AKT5 (n = 19), YFP+CBL9+CIPK10 (n = 17), YFP+AKT5+CBL9+CIPK10 (n = 29). Statistical significance was examined using Tukey-Kramer test (*$p < 0.05$).

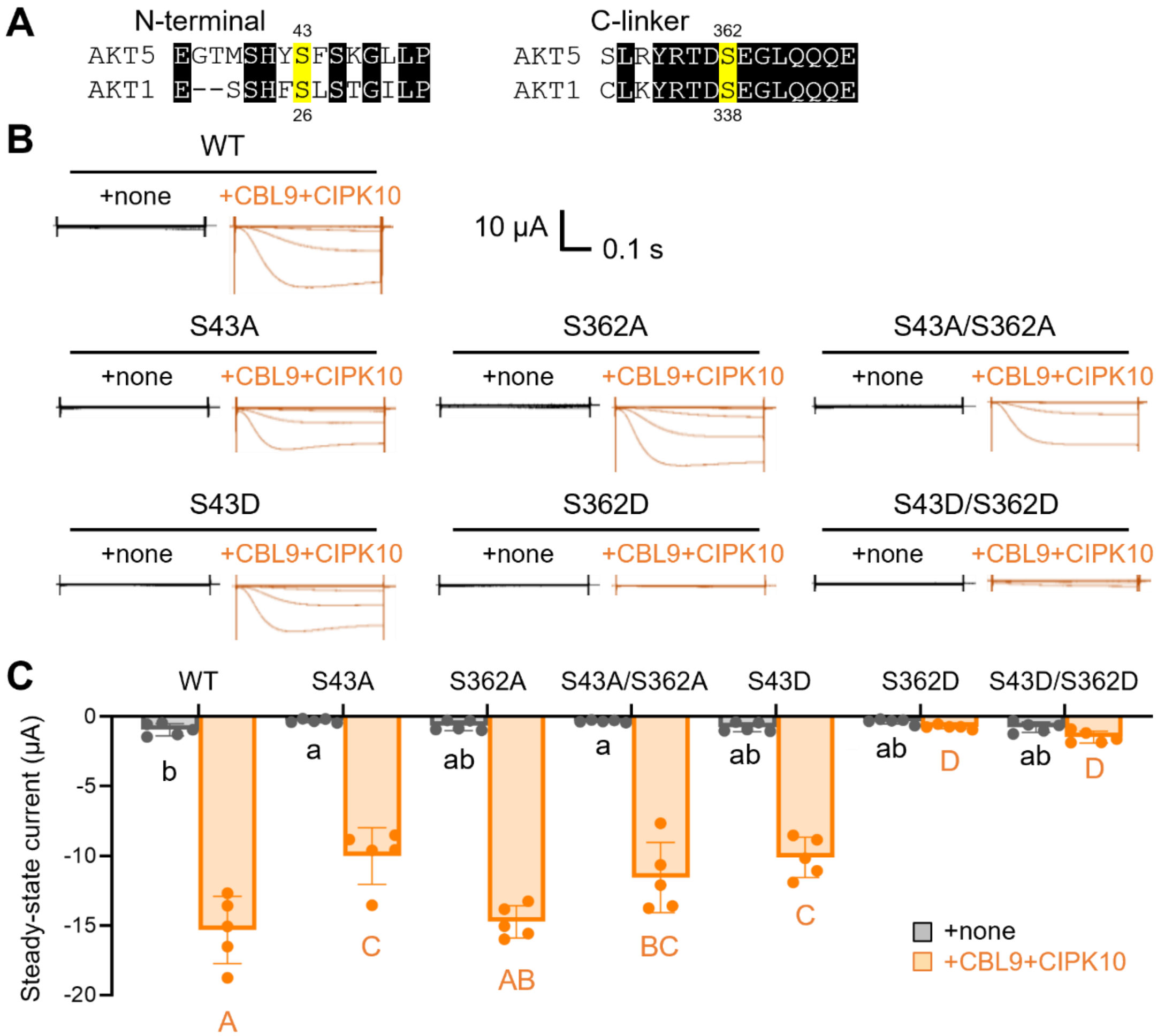

Fig. S9. Investigation of potential phosphorylatable serines in AKT5.

(**A**) Two serine residues (S26 and S338) in AKT1 targeted by CBL1-CIPK23 are conserved in AKT5. (**B** and **C**) Electrophysiological recordings of AKT5 variants carrying replacement of S43 and/or S362 expressed in *Xenopus* oocytes. Currents were elicited by voltage steps from +10 to −190 mV (Δ = −20 mV). Currents were recorded in standard buffers containing 120 mM K^+. Representative current profiles of AKT5 variants (B). Images were cropped to display only the relevant regions. (C) Steady-state currents recorded at −190 mV for AKT5 variants shown in (B). Data are presented as mean ± SD (n = 5). Different letters indicate statistically significant differences (one-way ANOVA with Tukey's multiple comparisons test; $p < 0.05$).

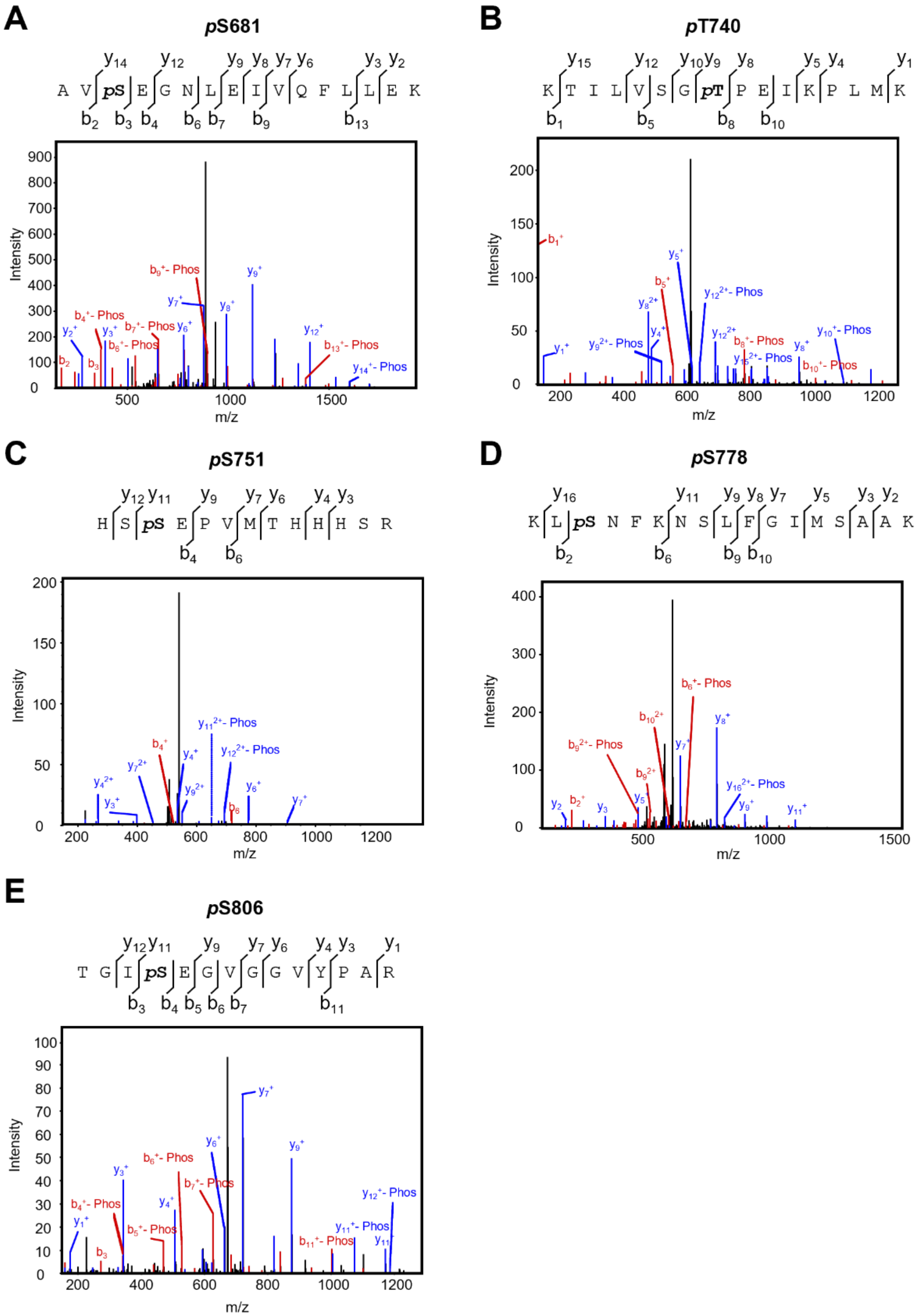

Fig. S10. MS/MS spectra of phosphorylated peptides derived from AKT5.

(**A**) MS/MS spectra corresponding to the phosphopeptides from AKT5 AVSEGNLEIVQFLLEK containing phospho-Ser681. (**B**) KTILVSGTPEIKPLMK containing phospho-Thr740. (**C**) HSSEPVMTHHHSR containing phospho-Ser751. (**D**) KLSNFKNSLFGIMSAAK containing phospho-Ser778. (**E**) TGISEGVGGVYPAR containing phospho-Ser806.

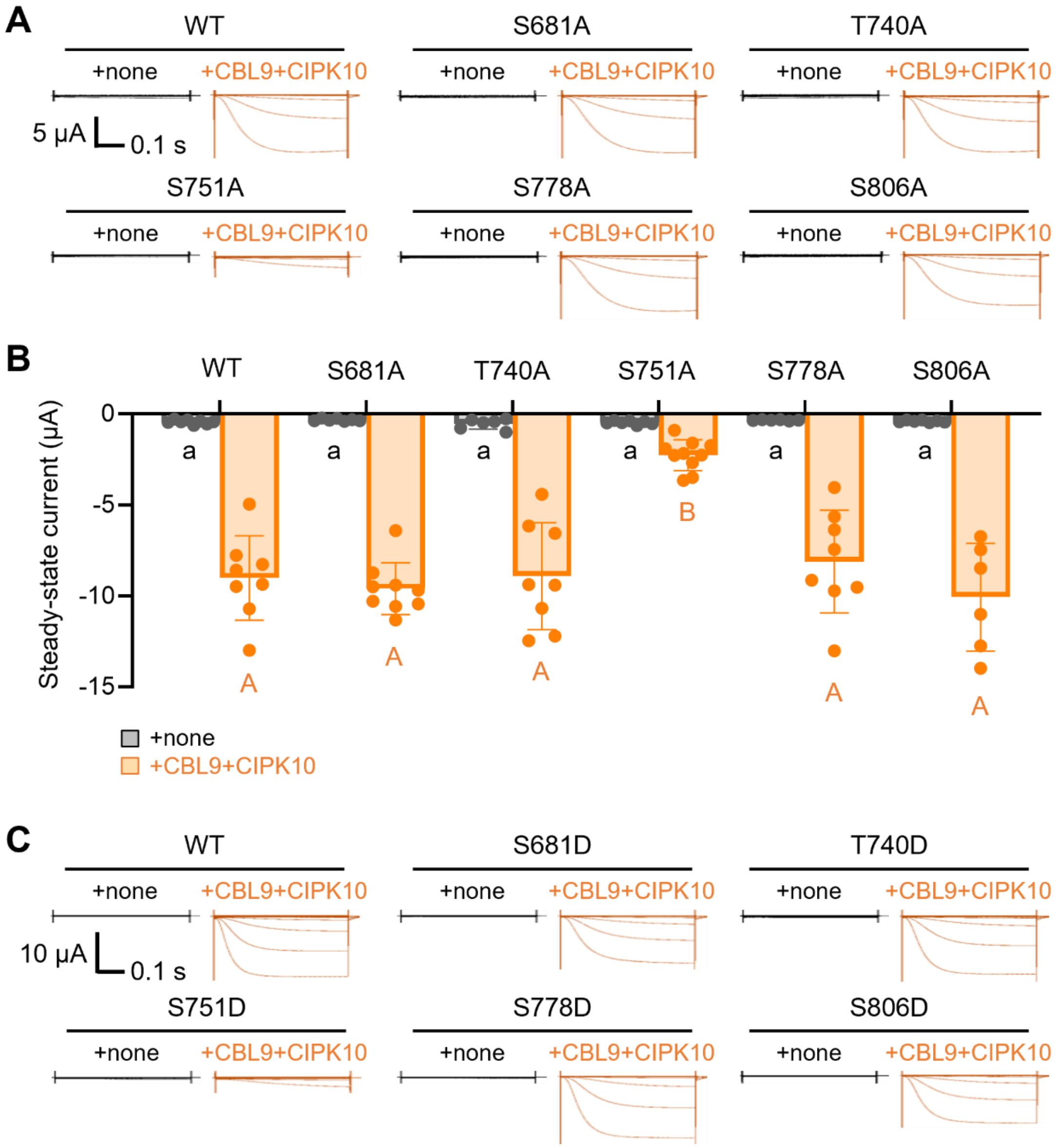

Fig. S11. Functional analysis of putative phosphorylatable Ser/Thr in AKT5 identified by mass spectrometry.

(**A**) Representative whole-cell current profile of AKT5 alanine-substituted variants (Ser/Thr to Ala) at phosphorylation sites identified by mass spectrometry, expressed in Xenopus oocytes. Currents were elicited by voltage steps from +10 to −190 mV (Δ = −20 mV). Images were cropped to show only the relevant regions. (**B**) Steady-state currents recorded at −190 mV for AKT5 variants shown in (A). Data are presented as mean ± SD (n = 6-10). Different letters indicate statistically significant differences (one-way ANOVA with Tukey's multiple-comparisons test; $p < 0.05$). (**C**) Electrophysiological recordings of AKT5 aspartate-substituted variants at phosphorylation sites identified by mass spectrometry expressed in Xenopus oocytes. Currents were elicited by voltage steps from +10 to −190 mV (Δ = −20 mV). Images were cropped to display only the relevant regions.

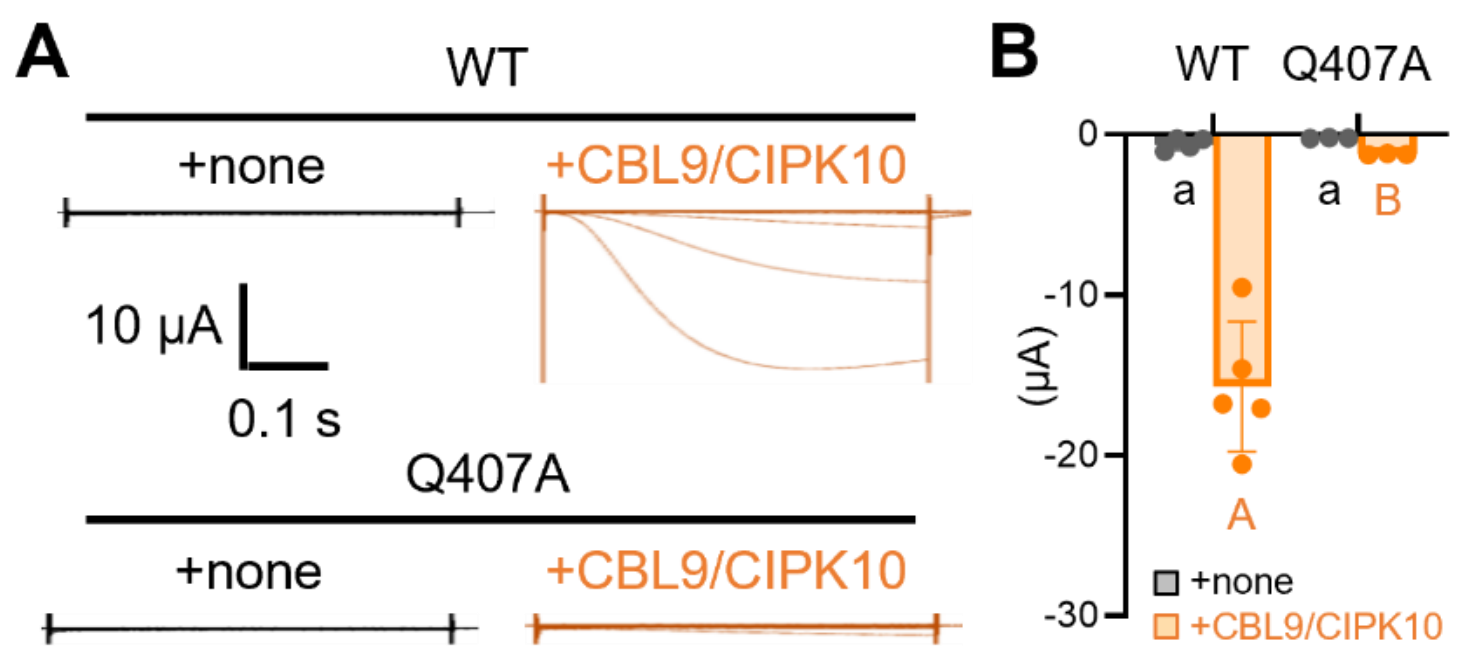

Fig. S12. Gln407 is also necessary for the full activation of AKT5.

(**A**) Representative current traces recorded from Xenopus oocytes expressing AKT5 (WT) and AKT5^{Q407A}, which was designed to disrupt the interactions at the CNBHD interfaces. Currents were elicited by voltage steps from +10 to −190 mV (Δ = −20 mV). Images were cropped to display only the relevant regions. (**B**) Steady-state currents recorded at −190 mV as shown in panel (A). The data are presented as mean ± SD (*n* = 3-5). Different letters indicate significant differences (ordinary one-way ANOVA with Tukey's multiple comparisons test; $p < 0.05$).

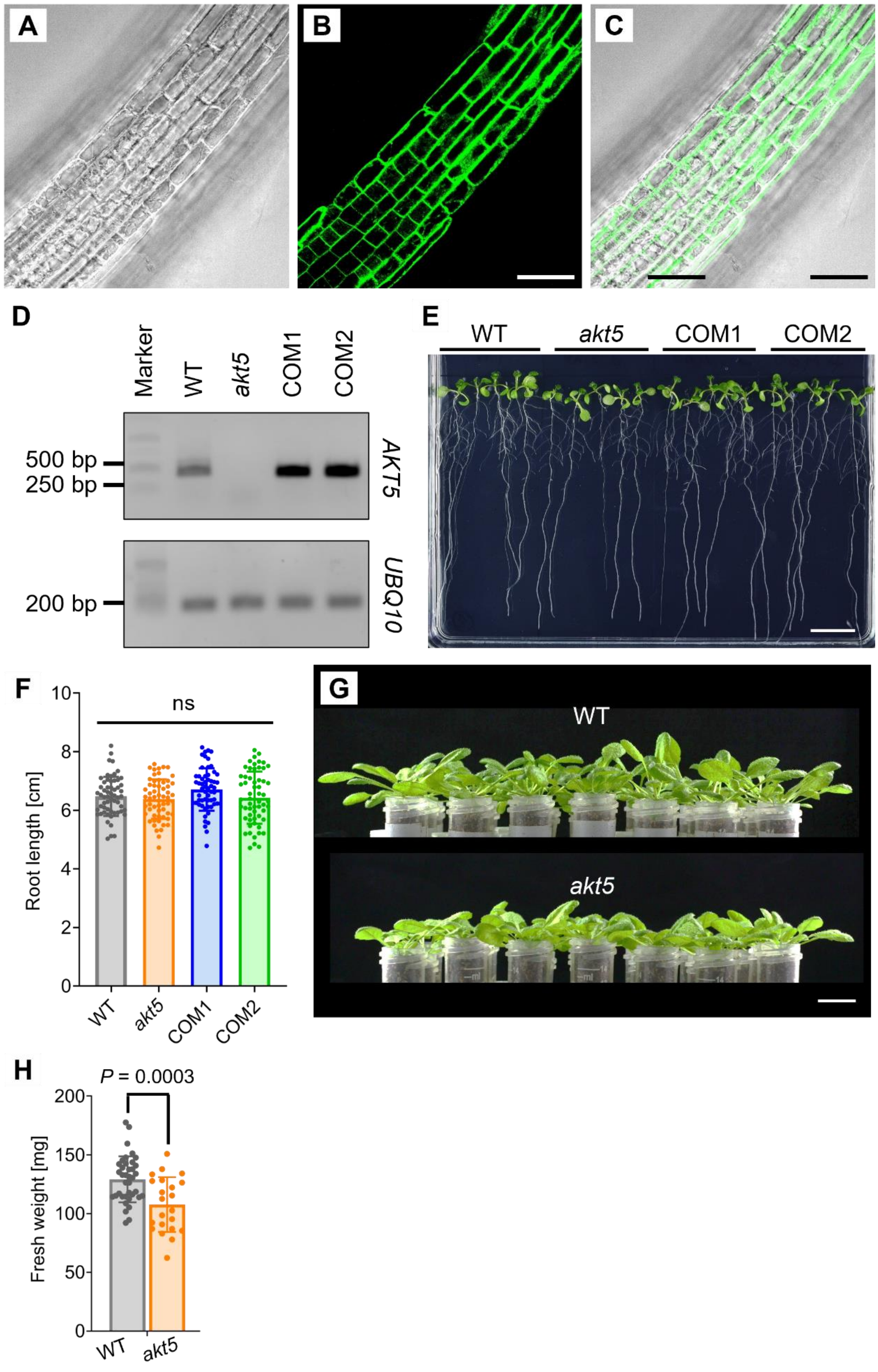

A
B
C
D
Marker
WT
akt5
COM1
COM2
500 bp
250 bp
200 bp
AKT5
UBQ10
E
WT
akt5
COM1
COM2
F
ns
Root length [cm]
0
2
4
6
8
10
WT
akt5
COM1
COM2
G
WT
akt5
H
P = 0.0003
Fresh weight [mg]
0
50
100
150
200
WT
akt5

Fig. S13. Phenotypic characterization of the *akt5* mutant and its complementation lines.

(**A**-**C**) Plasma membrane localization of ATK5 in *Arabidopsis thaliana*. GFP-tagged AKT5 expressed in root cells (*P_{35S}-GFP-AKT5*) was visualized by confocal microscopy. Figure 4A presents a portion of this photograph. (**A**) Bright field image, (**B**) GFP fluorescence, (**C**) merged image. Scale bar = 50 μm. (**D**) RT-PCR analysis using cDNA synthesized from total mRNA isolated from whole seedlings of 9-day-old *Arabidopsis*. The AKT5 PCR product was detected in all genotypes except the *akt5* mutant. UBQ10 was used for a positive control. (**E**) Representative photograph of 9-day-old *Arabidopsis* seedlings grown on 1/2 MS medium. Scale bar = 2 cm. (**F**) Root length of 9-day-old *Arabidopsis* seedlings grown on 1/2 MS medium. Data are presented as mean ± SD (n = 58-59). No significant differences were detected (one-way ANOVA with Tukey's multiple comparisons test; $p > 0.05$). (**G**) Representative images depicting the crowded growth conditions of the WT (Col-0, top) and the *akt5* mutant (bottom) grown in 15-ml tubes. The tube volume differs from that conducted in Fig. 4K and L. Scale bar = 2 cm. (**H**) Fresh weight of WT and the *akt5* mutant shown in (G). Data are presented as mean ± SD (n = 38 for WT, 22 for the *akt5* mutant). Statistical significance was assessed using unpaired two-tailed t-test.

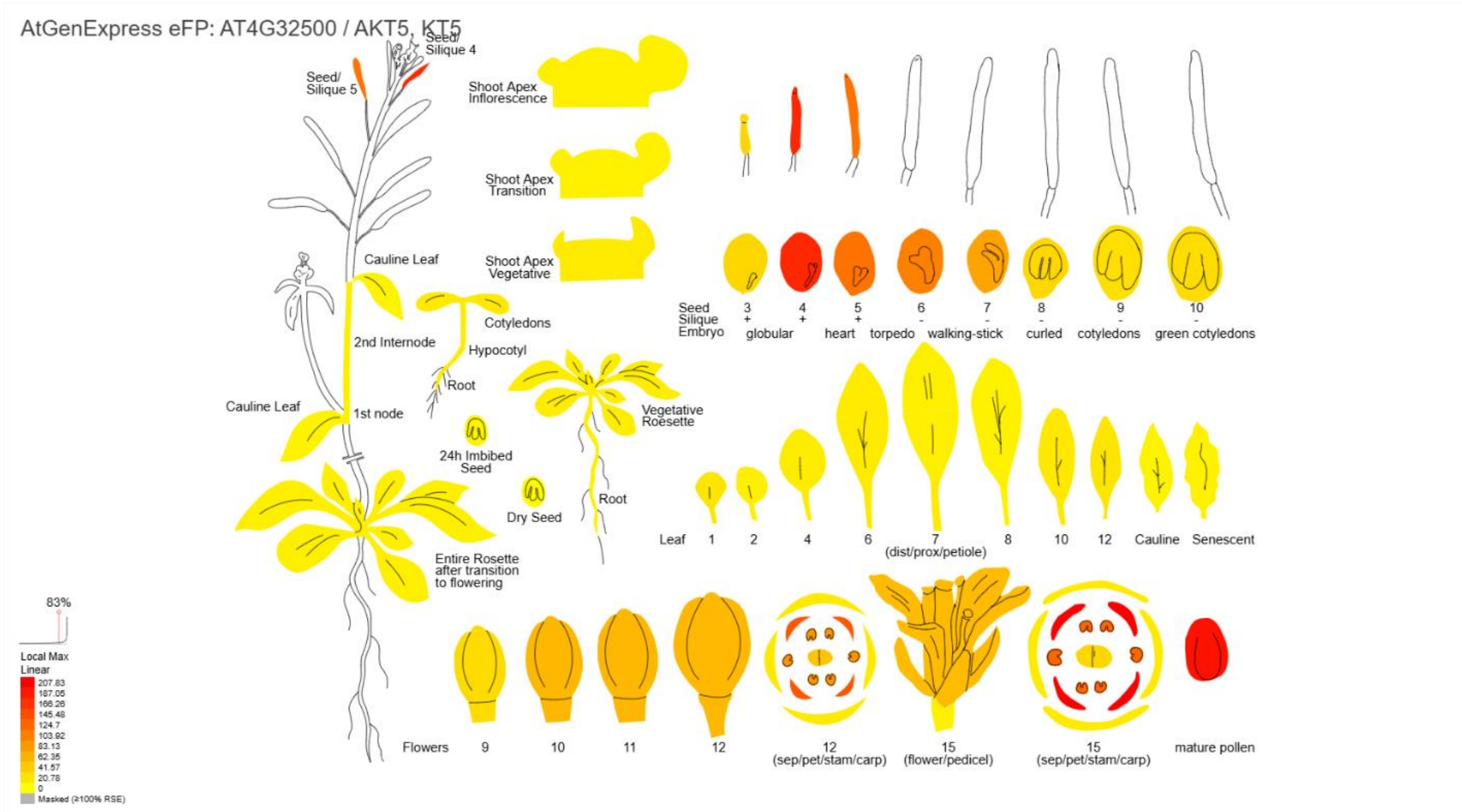

Fig. S14. Tissue-specific expression pattern of *AKT5* (AT4G32500) from the ePlant.

Expression pattern of *AKT5* (AT4G32500) based on publicly available transcriptome data from the ePlant (https://bar.utoronto.ca/eplant). Color scale represents expression levels (Local Max Linear, 0–207.83).

**Dataset**	AKT5 - WT (EMDB-66431) (PDB 9X0B)	AKT5 - D403A (EMDB-66432) (PDB 9X0C)
**Data collection and processing**		
Magnification	60,000	60,000
Voltage (kV)	300	300
Electron exposure (e–/Å^2)	60	60
Defocus range (μm)	-0.7 to -2.0	-0.6 to -2.2
Pixel size (Å)	0.806	0.825
Symmetry	C2	C4
Final particle images (no.)	54,571	186,912
Map resolution (Å)	3.62	2.60
FSC threshold	0.143	0.143
**Refinement**		
Map sharpening B factor (Å^2)	-97.5	-82.6
Model composition		
Non-hydrogen atoms	12,726	14,592
Protein residues	1,626	1,796
Protein B factors (Å^2)	33.90	69.93
R.m.s. deviations		
Bond lengths (Å)	0.013	0.013
Bond angles (°)	1.496	1.561
Validation		
MolProbity score	2.94	2.86
Clashscore	14.66	13.23
Ramachandran plot		
Favored (%)	92.09	93.18
Allowed (%)	7.78	6.77
Outliers (%)	0.12	0.06
Rotamer outliers (%)	10.85	10.94

Table S1. Cryo-EM data acquisition, refinement and validation statistics.

Movie S1. Interaction changes at the CNBHD interfaces during the C2- to C4 conformational transition of AKT5.

In C2-symmetric conformation, Y471 (green) in Mol II/II’ interacts with Q407 (red) in Mol I/I’ and G449 (green) in Mol I/I’ interacts with Q407 (red) in Mol II/II’. In the C4-symmetric conformation, N427 (blue) interacts with Q407 (red) at four sites.

Movie S2. Petiole elongation of *Arabidopsis thaliana* recorded by time-lapse video imaging.

Two-week-old plants were grown in soil and imaged under dim green light (0.03 μmol/m^2/s). The three pots were arranged from left to right: WT (Col-0), *akt5* mutant, complemented line (COM2). The clock represents zeitgeber time in the growth chamber.
